# PI3Kβ inhibition enhances ALK‐inhibitor sensitivity in *ALK* ‐rearranged lung cancer

**DOI:** 10.1002/1878-0261.13342

**Published:** 2023-01-25

**Authors:** Sarang S. Talwelkar, Mikko I. Mäyränpää, Julia Schüler, Nora Linnavirta, Annabrita Hemmes, Simone Adinolfi, Matti Kankainen, Wolfgang Sommergruber, Anna‐Liisa Levonen, Jari Räsänen, Aija Knuuttila, Emmy W. Verschuren, Krister Wennerberg

**Affiliations:** ^1^ Institute for Molecular Medicine Finland (FIMM), HiLIFE University of Helsinki Finland; ^2^ Institute of Biomedicine and MediCity Research Laboratories University of Turku Finland; ^3^ Department of Pathology Helsinki University Hospital and University of Helsinki Finland; ^4^ Charles River Research Services Germany GmbH Freiburg im Breisgau Germany; ^5^ A. I. Virtanen Institute for Molecular Sciences University of Eastern Finland Kuopio Finland; ^6^ Cancer Cell Signalling Boehringer Ingelheim RCV GmbH & Co KG Vienna Austria; ^7^ Department of Biotechnology University of Applied Sciences Vienna Austria; ^8^ Department of Thoracic Surgery, Heart and Lung Center Helsinki University Hospital Finland; ^9^ Department of Pulmonary Medicine, Heart and Lung Center and Cancer Center Helsinki University Hospital Finland; ^10^ iCAN Digital Precision Cancer Medicine Flagship Finland; ^11^ Translational Cancer Medicine Research Program, Faculty of Medicine University of Helsinki and Helsinki University Hospital Finland; ^12^ Biotech Research & Innovation Centre (BRIC) and Novo Nordisk Foundation Center for Stem Cell Biology (DanStem) University of Copenhagen Denmark

**Keywords:** *ALK*‐rearranged lung cancer, combination treatment, drug resistance, EGFR, EML4‐ALK, NSCLC, patient‐derived cells, PI3Kβ

## Abstract

Treatment with anaplastic lymphoma kinase (ALK) inhibitors significantly improves outcome for non‐small‐cell lung cancer (NSCLC) patients with *ALK*‐rearranged tumors. However, clinical resistance typically develops over time and, in the majority of cases, resistance mechanisms are ALK‐independent. We generated tumor cell cultures from multiple regions of an *ALK*‐rearranged clinical tumor specimen and deployed functional drug screens to identify modulators of ALK‐inhibitor response. This identified a role for PI3Kβ and EGFR inhibition in sensitizing the response regulating resistance to ALK inhibition. Inhibition of ALK elicited activation of EGFR, and subsequent MAPK and PI3K‐AKT pathway reactivation. Sensitivity to ALK targeting was enhanced by inhibition or knockdown of PI3Kβ. In *ALK*‐rearranged primary cultures, the combined inhibition of ALK and PI3Kβ prevented the EGFR‐mediated ALK‐inhibitor resistance, and selectively targeted the cancer cells. The combinatorial effect was seen also in the background of *TP53* mutations and in epithelial‐to‐mesenchymal transformed cells. In conclusion, combinatorial ALK‐ and PI3Kβ‐inhibitor treatment carries promise as a treatment for *ALK*‐rearranged NSCLC.

## Graphical abstract

PI3Kβ inhibitors enhance the response to ALK inhibitors in primary *ALK*‐rearranged lung cancer cells. PI3Kβ inhibition blocks ALK‐inhibition‐induced cytoprotective reactivation of EGFR signaling, enhancing the cytotoxic effect on both epithelial‐ and mesenchymal‐phenotype cancer cells without affecting normal lung epithelial cells. Combined inhibition of both ALK and PI3Kβ therefore represents a promising approach to improve clinical responses in *ALK*‐rearranged lung cancers.

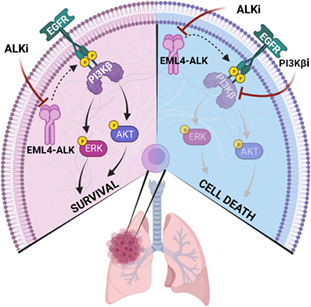

**Abbreviations**
AKT: RAC‐alpha serine/threonine‐protein kinase
ALK: anaplastic lymphoma kinase
ALKi: ALK inhibitor
ATCC: American Type Culture Collection
AUC: area under the curve
AXL: AXL receptor tyrosine kinase
BCA: bicinchoninic acid
CBFA2T3: CBFA2/RUNX1 partner transcriptional co‐repressor 3
CD31: cluster of differentiation 31
Co‐IP: Co‐immunoprecipitation
CR: conditional reprogramming
DMEM: Dulbecco's Modified Eagle Medium
DMSO: dimethyl sulfoxide
DSRT: drug sensitivity and resistance testing
DSS: drug sensitivity score
EGF: epidermal growth factor
EGFR: epidermal growth factor receptor
EML4: echinoderm microtubule‐associated protein‐like 4
EMT: epithelial–mesenchymal transition
ERBB: Erb‐b2 receptor tyrosine kinase
ERK: extracellular signal‐regulated kinase
FBS: fetal bovine serum
FISH: fluorescence *in situ* hybridization
GDSC: genomics of drug sensitivity in cancer
GPCR: G protein‐coupled receptor
H&E: hematoxylin and eosin
IC50: half‐maximal inhibitory concentration
IGF‐1R: insulin‐like growth factor‐1 receptor
IgG: immunoglobulin G
IHC: immunohistochemistry
KIT: KIT proto‐oncogene
KRAS: Kirsten rat sarcoma viral oncogene homolog
MAPK: mitogen‐activated protein kinase
MET: MET proto‐oncogene, receptor tyrosine kinase
mTOR: mechanistic target of rapamycin kinase
NFE2L2: nuclear factor erythroid‐derived 2‐like 2
NKX2.1: NK2 homeobox 1
NMRI: naval medical research institute
ns: not significant
NSCLC: non‐small cell lung cancer
NTC: non‐targeting control
P2Y: purinergic receptor P2Y1
PARP: poly‐(ADP‐ribose) polymerase
PDX: patient‐derived xenograft
PI3K: phosphoinositide 3‐kinase
PI3Kβ: phosphoinositide 3‐kinase β
PIK3CA/PI3Kα: phosphatidylinositol‐4,5‐bisphosphate 3‐kinase catalytic subunit alpha
PIK3CB/PI3Kβ: phosphatidylinositol‐4,5‐bisphosphate 3‐kinase catalytic subunit beta
PPP2R1A: protein phosphatase 2 scaffold subunit alpha
PTEN: phosphatase and tensin homolog
qRT‐PCR: quantitative real‐time polymerase chain reaction
RIPA: radioimmunoprecipitation assay
ROCK: rho‐associated protein kinase
RTK: receptor tyrosine kinase
SEM: standard error of the mean
SHP2: Src homology region 2 domain‐containing phosphatase‐2
siRNA: small interfering RNA
SMARCA4: SWI/SNF related, matrix associated, actin dependent regulator of chromatin, subfamily a, member 4
SRC: SRC proto‐oncogene, non‐receptor tyrosine kinase
STR: short tandem repeat
TP53: tumor protein P53
TR: tumor region

## 1 Introduction

In 3–7% of NSCLCs, *ALK* gene rearrangements lead to the expression of oncogenic fusion proteins that confer constitutive activity of the ALK kinase domain [1, 2]. Aberrant ALK activity, in turn, activates the MAPK and PI3K‐AKT oncogenic signaling pathways [3, 4]. Treatment of *ALK*‐rearranged NSCLC with first‐ or second‐generation ALK inhibitors associates with favorable initial response in the majority of patients, although the development of resistance and clinical relapse typically occurs within a few years [5]. Five to 10% of patients show progressive disease following alectinib treatment, at present the recommended first‐line therapy for *ALK*‐rearranged NSCLC [5, 6]. Intrinsic resistance may relate to, for example, the precise *ALK* fusion variant [7] or *TP53* mutations which frequently co‐occur with *ALK* rearrangements [8, 9, 10]. Among tumors that acquire resistance to ALK inhibitors, 30–50% exhibit ALK‐dependent resistance mechanisms, particularly *ALK* mutations and/or amplification [11, 12]. In the remainder, resistance associates with diverse mechanisms, including activation of bypass signaling pathways (e.g., EGFR, KIT, IGF‐1R, or GPCRs), epithelial‐to‐mesenchymal transition (EMT), a histopathology switch, or increased activity of drug efflux pumps [3].

For *ALK*‐rearranged NSCLCs exhibiting ALK‐dependent acquired resistance, second‐ or third‐generation ALK inhibitors that retain activity in the presence of resistance mutations to first‐line inhibitors offer optional “sequential” treatment opportunities. However, the mechanistic drivers for resistance, such as bypass signaling, are not diagnostically assessed and likely differ between samples. In addition to pronounced intertumor variability in resistance mechanisms, *ALK*‐rearranged lung tumors also exhibit intratumor heterogeneity in ALK inhibitor resistance [3]. To address this clinical problem, patient‐derived cultures from ALK inhibitor‐resistant tumors can be used to identify treatment‐adaptive resistance mechanisms and combinatorial treatment approaches [13, 14, 15]. Nevertheless, as of yet, no combinatorial treatments to counter bypass signaling are available in the clinic.

Here, we performed multiregional characterization of an aggressive chemoresistant *ALK*‐rearranged lung tumor and corresponding tumor‐derived cultures. Drug response profiles were different in cultures derived from a region exhibiting an epithelial‐to‐mesenchymal transition (EMT) phenotype. Importantly, our study identified PI3Kβ as a novel target for improving response to ALK inhibition, and combinatorial ALK and PI3Kβ inhibitory response was detected in all cultures, also in the context of EMT and *TP53* mutation. As PI3Kβ is an effector of multiple tyrosine kinase activities that can mediate ALK inhibitor resistance, including EGFR, the co‐targeting of PI3Kβ and ALK offers promise as a treatment for *ALK*‐rearranged lung cancer.

## 2 Materials and methods

### 2.1 Culture of patient‐derived cells and cell lines

Procedures conducted in this study were performed in accordance with licenses approved by the Coordinating Ethics Committee of the University of Helsinki (85/13/03/00/2015, HUS/1204/2019). Clinical samples were collected at the Helsinki University Central Hospital (HUCH) with the patient's written informed consent. The patient samples used in this study were obtained from PLT26. In 2015, when the ALK rearrangement was identified in this patient's tumor, chemotherapy was the first‐line therapy at HUCH in Finland. The patient was therefore never treated with ALK‐targeted therapy. Immediately after surgery, a thoracic pathologist dissected the tumor and tumor‐adjacent normal lung tissues. Tissue pieces were manually minced using a sterile scalpel, and then enzymatically digested using the Tumor Dissociation Kit (Miltenyi, Bergisch Gladbach, Germany; 130‐095‐929) and gentleMACS Dissociator (130‐093‐235), following the manufacturer's instructions. Single cell suspensions were further processed using Miltenyi's human EpCAM (CD326) cell isolation kit (130‐061‐101) to enrich EpCAM^+^ cells. Both normal and tumor EpCAM+ cells were cultured in the presence of irradiated (30 Gy) 3T3 feeder cells in F‐medium using Conditional Reprogramming (CR) culture protocol as described in [16, 17]. Feeder 3T3 cells were cultured in DMEM (Thermo Fisher Scientific, Waltham, MA, USA; 42430‐025) supplemented with 10% FBS (Gibco, Waltham, MA, USA; 10270‐106). Differential trypsinization was utilized for culture propagation when cells reached 80% confluency [16]. Lung cancer cell lines (H3122, H2228, A549, H460, Calu‐1, and H1437) were cultured in RPMI‐1640 (Lonza, Basel, Switzerland; 15‐1675) supplemented with 10% FBS. All cultures were maintained in a humidified incubator at 37 °C with 5% CO_2_. H3122 (Cellosaurus accession ID: CVCL_5160), H2228 (CVCL_1543), A549 (CVCL_0023), H460 (CVCL_0459), Calu‐1 (CVCL_0608), and H1437 (CVCL_1472) cells were purchased from the ATCC (Manassas, VA, USA) and STR‐profiled at the Genotyping Unit of Technology Centre at the Institute for Molecular Medicine Finland (FIMM).

### 2.2 F‐medium

F‐medium is a combination of 1:3 v/v DMEM: F‐12 nutrient HAM supplemented with 5% FBS, 10 ng·mL^−1^ EGF (BD Biosciences, Franklin Lakes, NJ, USA; 354 052), 5 μg·mL^−1^ insulin (Sigma, St. Louis, MO, USA; I2643), 24 μg·mL^−1^ adenine (Sigma; A2786), 0.4 μg·mL^−1^ hydrocortisone (Sigma; H4001), 10 ng·mL^−1^ cholera toxin (List Biological Laboratory, Campbell, CA, USA; 100B), and 10 μm ROCK inhibitor (Y‐27632; ENZO) and penicillin–streptomycin (Gibco; 15140‐122) to final concentrations of 100 Units·mL^−1^ and 100 μg·mL^−1^, respectively.

### 2.3 Drug sensitivity and resistance testing (DSRT) assay

Drug sensitivity and resistance testing assays for anticancer compounds as a single agent or in combination with other compounds were performed as previously described [17]. For drug screening, 384‐well plates (Corning, Corning, NY, USA; 3712) with compounds were prepared in advance by dispensing compounds using an Echo 550 liquid handler (Labcyte, Synnyvale, CA, USA), at five concentrations covering a 10 000‐fold concentration range. For storage, the predrugged plates were kept in pressurized StoragePods (Roylan Developments Ltd., Fetcham, UK) under inert nitrogen gas. For drug screening, the predrugged compounds were dissolved in 5 μL of culture medium per well, with (1 : 2000 final volume) or without CellTox Green (Promega, Madison, WI, USA) depending on the experiment, and 20 μL cell suspension per well was seeded at a concentration of 1500 cells per well. After 72‐h incubation at 37 °C, cell death was assessed by measuring fluorescence signals from CellTox Green (485/520 nm excitation/emission filters). Subsequently, to assess cell viability, 25 μL per well CellTiter‐Glo reagent (Promega) was added, and luminescence was recorded. Both fluorescence and luminescence readouts were recorded using a PHERAStar FS plate reader (BMG Labtech, Ortenberg, Germany). To plot dose–response curves for each drug, the Marquardt–Levenberg algorithm was implemented using the in‐house‐developed bioinformatic ‘Breeze’ pipeline [18]. To compare drug responses across samples, Drug Sensitivity Scores (DSSs) were calculated using dose–response curve parameters including the IC50, slope, top, and lower asymptotes, as described [19]. To manually assess the drug responses of single agents or drug combinations, a denser concentration range (nine doses between 0.5/1 and 5000/10 000 nm) of compounds was used. Cells (1500 per well) were seeded in 384‐well plates in 20 μL of media. After 24‐h incubation at 37 °C, cells were treated with vehicle control or drug in 10 μL of media with three technical replicates for each condition. After 72‐h incubation at 37 °C, cell viability was quantified using CellTiter‐Glo reagents. The relative cell viability was calculated using the formula: (cell viability of drug treatment)/(cell viability of vehicle control) × 100. Values for the selectivity index are calculated using the formula p(IC50 *ALK*‐rearranged cells/IC50 *ALK* wildtype cells).

### 2.4 Immunoblotting

Lysates were prepared from tumor tissue or cultured cells using RIPA buffer supplemented with fresh protease and phosphatase inhibitors (Roche, Penzberg, Germany). Protein quantification was performed using the BCA Protein Assay (G Biosciences, St. Louis, MO, USA; 786‐570). Lysates were fractionated using Mini‐PROTEAN TGX precast gels (Biorad, Hercules, CA, USA) and transferred to PVDF membranes (Millipore, Burlington, MA, USA; IPFL00010) using the XCell II blot module (Thermo‐Scientific). After transfer, membranes were blocked for 30 min at room temperature using Odyssey Blocking Buffer. Two‐color immunoblotting was performed using Odyssey Blocking Buffer (LI‐COR, Lincoln, NE, USA) and IRDye (800CW/680RD) secondary antibodies (LI‐COR) diluted 1:10 000 in Odyssey Blocking Buffer. Membranes were scanned using an Odyssey infrared imager (LI‐COR) and quantifications were performed using the Image Studio software (LI‐COR). Primary antibodies are listed in the Supplemental Experimental Procedures. Table S1 in the Supporting Information section lists the antibodies and dilutions used for immunoblotting.

### 2.5 Co‐immunoprecipitation

Co‐immunoprecipitation (Co‐IP) experiments were performed using the Thermo Fisher Scientific Pierce Co‐IP Kit (26149), following the manufacturer's protocol. In brief, cells were exposed to drugs when cell confluency reached 60–70%. After 4 or 24 h of drug exposure, cells were lysed with an ice‐cold Lysis/Wash Buffer supplemented with fresh protease and phosphatase inhibitors (Roche). Cell lysates were precleared by incubating the lysate with Control Agarose Resin at 4 °C for 1 h. To prepare antibody‐immobilized AminoLink Plus Coupling Resin, 25 μL Coupling Resin, and 1 μg of rabbit anti‐EGFR (Santa Cruz Biotechnology, Dallas, TX, USA; sc‐120) or IgG control antibody (Invitrogen, Waltham, MA, USA; 02‐6102) were co‐incubated in a spin column for 2 h at room temperature. Columns were then washed twice with 1× Coupling Buffer and six times with Wash Solution, and centrifuged after each wash. Subsequently, precleared cell lysate was added to the column containing antibody‐coupled resin, and incubated overnight at 4 °C with gentle mixing. Proteins were eluted using 60 μL of Elution Buffer, and eluates were analyzed by western blotting with primary antibodies for EGFR, pEGFR, PI3Kβ, and tubulin. See Supplementary Information section for antibody details. Table S1 in the Supporting Information section lists the antibodies and dilutions used for Co‐IP.

### 2.6 IncuCyte confluency assay

Cells were seeded at a density of 5000–10 000 cells per well in 96‐well plates and were exposed to drugs or DMSO on the following day. Immediately after drug treatment, cells were followed by live‐cell imaging using an IncuCyte ZOOM microscope (Essen Bioscience, Ann Arbor, MI, USA) by taking pictures every 3 or 4 h for a total of 72 or 120 h. Images were then quantified for cell confluency (cell surface area coverage as confluence values) using the incucyte application software (Essen Bioscience), and cell confluency data were plotted with GraphPad Prism (GraphPad Software, San Diego, CA, USA).

### 2.7 Colony formation assay

Cells were seeded at a density of 10 000–20 000 cells per well in 24‐well plates and 50 000 cells per well in six‐well plates. On the following day, cells were exposed to drugs or DMSO. Medium and DMSO/drugs were replaced every 72 h for 15 days. Cells were fixed with colony fixation solution [acetic acid/methanol 1 : 7 (vol/vol)] and stained with 0.5% crystal violet. After drying, stained plates were scanned with the Cytation 5 Cell Imaging Multi‐Mode Reader (BioTek, Winooski, VT, USA) and the number and size of colonies were quantified with the compatible Gen5™ Multi‐Mode Reader and Imager Software (BioTek). To assess the long‐term effect of ALK inhibitors, cells were treated with IC_50_ doses of each ALK inhibitor that were calculated based on 3‐day drug response measurements.

### 2.8 Autophagy analysis

Cells were seeded at a density of 10 000–20 000 cells per well in 96‐well plates and exposed to drugs or DMSO on the following day. Following 24‐h incubation, cells were costained with CYTO‐ID Green detection reagent and Hoechst 33342 nuclear stain according to the manufacturer's instructions (Enzo Life Science, San Diego, CA, USA). Cells were observed and imaged using Opera Phenix High‐Content Screening System (PerkinElmer, Waltham, MA, USA). Quantification of the number of autophagic vacuoles, vacuoles per cell, their size, and the intensity of the vacuoles was carried out using the system's hharmony software (PerkinElmer).

### 2.9 Drug treatment with or without EGF

Cells (5000 cells per well) were cultured in 96‐well plates in media without EGF. After 24 h, cells were treated with indicated drug concentration or vehicle control in media supplemented with 10 or 100 ng·mL^−1^ fresh EGF, or without addition of EGF. Drug and EGF treatments were repeated every 24 h for 3 days. After 72 h of total drug treatment, cell viability was quantified using CellTiter‐Glo reagents. The relative cell viability was calculated using the formula: (cell viability of drug treatment)/(cell viability of vehicle control) × 100.

### 2.10 *In vivo* treatment experiment

Treatment studies on patient‐derived xenograft (PDX) models derived from primary TR3 and TR5 ALK‐rearranged NSCLC cultures were performed at the Charles River facilities. This study was carried out in strict accordance with the recommendations in the Guide for the Care and Use of Laboratory Animals of the Society of Laboratory Animals (GV SOLAS) in an AAALAC accredited animal facility. All animal experiments were approved by the Committee on the Ethics of Animal Experiments of the regional council (Regierungspräsidium Freiburg, Abt. Landwirtschaft, Ländlicher Raum, Veterinär‐ und Lebensmittelwesen ‐ permit # G‐18/12). NSG (NOD.Cg‐Prkdcscid Il2rgtm1Wjl/SzJ) animals were used for *in vivo* treatment experiments, and animals were sourced from Charles River, France. Both male and female NSG mice (6–8 weeks old) were implanted subcutaneously with fragments from either TR3 or TR5 PDX tumors. Once average tumor volume reached 100 mm^3^, animals were randomized to different treatment groups (*n* = 5 per treatment group) and were administered with vehicle control, ceritinib alone, AZD‐8186 alone or combination of ceritinib plus AZD‐8186. Ceritinib was administered at 25 mg·kg^−1^ body weight p.o. daily for 21 days. AZD‐8186 was administered at 2 × 25 mg·kg^−1^ body weight p.o. daily for 21 days. Ceritinib (25 mg·kg^−1^) and AZD‐8186 (2 × 25 mg·kg^−1^) were administered together p.o. daily for 21 days. The control vehicle (0.5% Methylcellulose, 0.5% Tween80) p.o. for 21 days. Tumors were measured using electronic calipers twice a week, and tumor volumes were calculated using the formula length × width^2^ × 0.52. Body weights were recorded in parallel to the tumor volume measurement. Animals were monitored daily for signs of morbidity and/or mortality.

### 2.11 Animal housing and handling

Animals were housed in individually ventilated cages (TECNIPLAST Sealsafe‐IVC System, TECNIPLAST, Hohenpeissenberg, Germany), depending on group size, either in type III or in type II long cages. They were kept under a 14L:10D artificial light cycle. The temperature inside the cages was maintained at 22–26 °C with a relative humidity of 45–65% and 60–65 air changes·h^−1^ in the cage. Dust‐free bedding consisting of aspen wood chips with approximate dimensions of 5 mm × 5 mm × 1 mm (ABEDD, LAB & VET Service GmbH, Vienna, Austria; LTE E 001) and additional nesting material were used. The cages including the bedding and the nesting material were changed weekly. The animals were fed autoclaved Teklad Global Extruded 19% Protein Rodent Diet from Envigo RMS SARL (Gannat, France) and had access to sterile filtered and acidified (pH 2.5) tap water that was changed twice weekly. Feed and water were provided *ad libitum*. All materials were autoclaved prior to use. Animals were routinely monitored at least twice daily on working days and at least once daily on weekends and public holidays. Routine monitoring included inspections for dead animals, assessment of animal welfare and tumor growth by observation, control of feed and water supply and of technical housing conditions. Any observed or suspected impairment of animal welfare was documented.

### 2.12 Statistics and reproducibility

graphpad prism 9 (GraphPad Software Inc) was used to generate all figures presented and to perform statistical analyses of experimental data. Statistical significance was assessed using a Student's *t* test and nonparametric Mann–Whitney test or Wilcoxon matched‐pairs signed rank test. *P*‐values > 0.05 were considered as statistically significant. Error bars indicate standard deviation or standard error of the mean. Pearson's correlation coefficients were used to assess the significance of correlations and displayed in the XY plots. DSRT, NGS, and IHC experiments were performed a single time, with biological replicates (*n* = 2–6). Immunoblot analyses including co‐immunoprecipitation, FISH, and validation of siRNA‐mediated *PIK3CB* knockdown were performed two times, with similar results. Follow‐up experiments including colony formation assay, IncuCyte confluency assay, CYTO‐ID based autophagy assay, and experiments evaluating the effect of *PIK3CB* knockdown, EGF treatment, GPCR inhibition or autophagy inhibition on responses of ALKi, PI3Kβi or combination of ALKi plus PI3Kβi were performed three times, with similar results. Xenograft experiments were performed once at Charles River by different experimenters.

### 2.13 Immunohistochemistry

Tissue processing and IHC procedures were performed essentially as described previously [17]. Antibodies and stain‐specific details are listed in the Supplemental Information section. To acquire whole slide scans of stained tissue sections, the Pannoramic 250 digital slide scanner (3DHISTECH, Budapest, Hungary) was used and the scanned TIFF images are exported using the Pannoramic Viewer (3DHISTECH). Table S1 in the Supporting Information section lists the antibodies and dilutions used for IHC.

### 2.14 Fluorescence *in situ* hybridization (FISH)

The *ALK*‐rearrangement status of the samples was evaluated by FISH using *ALK* dual‐color break‐apart probe according to the manufacturer's protocol (Vysis, Abbott Molecular Inc., Abbott Park, IL, USA). Tumor cell nuclei with split red and green signals were defined as positive for *ALK‐*rearrangement, and at least 100 cells were evaluated for each sample to conclude the *ALK*‐rearrangement status. Samples displaying *ALK*‐rearrangement in more than 10% of the cells were labeled as positive.

### 2.15 Genetic analysis

Genomic DNA was extracted from healthy lung and tumor tissue samples and from corresponding CR cultures using a DNeasy Blood & Tissue kit (Qiagen, Hamburg, Germany). Targeted next‐generation sequencing was performed using the NimbleGen Cancer Panel (captures the exons of 578 cancer‐related genes; Roche Nimblegen, Madison, WI, USA) and the Illumina HiSeq2500 system in HiSeq high output mode using v4 chemistry or HiSeq Rapid run mode using v2 chemistry (Illumina, San Diego, CA, USA), as described [20]. Variants were removed if the variant allele frequency was < 2%; tools employed for variant calling have been outlined earlier [21].

### 2.16 *PIK3CB* RNAi and ALKi drug screens

*PIK3CB* gene silencing was achieved by RNA interference using a reverse transfection procedure. 384‐well plates (Corning; 3712) with siRNAs were prepared in advance by dispensing siRNAs using an Echo 550 liquid handler (Labcyte). Plates were either used immediately or foil‐sealed and stored at −20 °C until use. ON‐TARGET plus SMART pool siRNA (Dharmacon, Lafayette, CO, USA) against *PIK3CB* was used (10, 20 or 40 nm). Before transfection, siRNA molecules were reconstituted in OptiMEM (5 μL per well) medium (Invitrogen) containing Lipofectamine RNAiMAX (25 nL per well; Life Technologies, Waltham, MA, USA) using a Multidrop™ Combi nL Reagent Dispenser (Thermo Fisher Scientific). After 30‐min incubation at RT on an orbital shaker, 20 μL cell suspension per well was seeded in the siRNA plates. After 24‐h incubation at 37 °C, cells were treated with DMSO or 200 nm ceritinib and further incubated for 72 h at 37 °C. Cell viability was measured using CellTiter‐Glo reagent as per the manufacturer's instructions. Cells transfected with 10 nm of non‐targeting siRNA (Qiagen) or AllStars Hs Cell Death Control siRNA (Qiagen) served as a negative or positive control. To verify the knockdown of *PIK3CB*, 1 × 10^5^ cells were seeded in each well of a six‐well plate, after 24 h, cells were transfected with 10 nm *PIK3CB* siRNA using Lipofectamine RNAiMAX according to the manufacturers' instruction. At 72 h after transfection, lysates were collected and subjected to immunoblotting analyses.

## 3 Results

### 3.1 Intratumor heterogeneity of an *ALK* ‐rearranged lung tumor

**Fig. 1:**
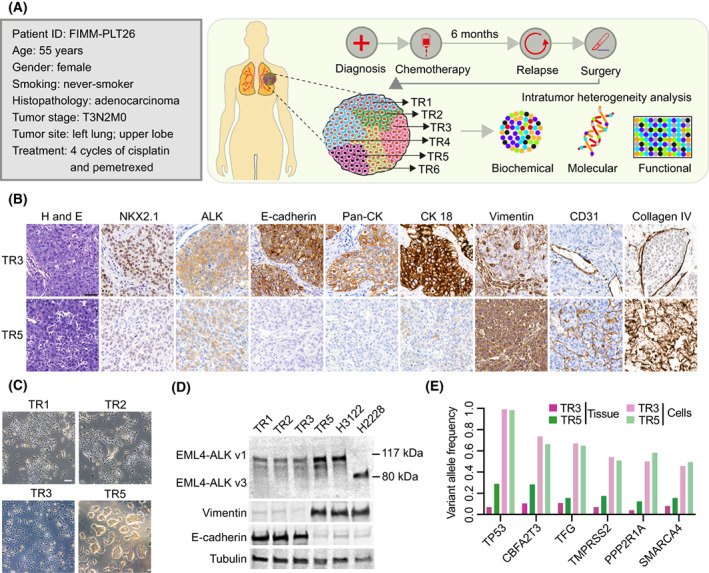
*ALK*‐rearranged lung tumor analysis reveals EMT‐related intratumor heterogeneity. (A) Clinical features and outline of intratumor heterogeneity analyses in a lung cancer specimen. (B) Representative images of hematoxylin and eosin (H&E) and IHC staining performed on TR3 and TR5 tissue regions (*n* = 1). Scale bar, 50 μm. (C) Representative bright field pictures of cultures derived from different tissue regions, all cultures with the exception of TR5 formed uniform epithelial monolayers (*n* = 3). Scale bar, 100 μm. (D) Immunoblots of *ALK*‐rearranged tumor‐derived cultures and cell lines, probed with the indicated antibodies. Lysates from H3122 and H2228 were used as a positive control for ALK variants 1 (v1) and 3 (v3), respectively (*n* = 1). (E) Variant allele frequencies of cancer‐selective somatic mutations identified in each sample (*n* = 1).

To capture the extent of intratumor heterogeneity and to identify treatments that can enhance sensitivity to ALK inhibition in *ALK*‐rearranged lung cancer, we characterized tumor tissues (*n* = 6) and cultures (*n* = 4) established from multiple tumor regions (TRs) of a large surgically resected tumor (9 × 12 × 9 cm) from a 55‐year‐old never‐smoker female patient with *ALK*‐rearranged T3N2M0 stage lung adenocarcinoma with diffuse metastasis. Before surgery, the patient was treated with chemotherapy, which did not halt disease progression (Fig. 1A and Fig. S1A,B). Immunohistochemical characterization of six tumor regions was done for ALK and markers of lung adenocarcinoma (NKX2.1), epithelial (E‐cadherin, pan‐cytokeratin, and cytokeratin 18) and mesenchymal (vimentin) phenotypes, tumor vasculature (CD31), and basement membrane organization (collagen type IV). All but one tissue region showed a similar phenotype with cancer cells exhibiting positivity for both NKX2.1 and the epithelial marker E‐cadherin. The exception was tumor region #5 (TR5), which exclusively showed aggressive lung cancer features, including aberrant basement membrane organization evidenced by enhanced collagen type IV staining, high tumor vasculature, and mesenchymal phenotype cancer cells lacking NKX2.1 expression (Fig. 1B and Fig. S1C). Importantly, TR5‐derived cells exhibited predominant expression of vimentin corroborating the parental mesenchymal tissue phenotype of the cancer cells in TR5. Unlike other TRs, TR5 cells expressed a high level of ALK fusion protein and formed multilayered tightly packed colonies in *ex vivo* culture (Fig. 1C,D and Fig. S1D). The EML4‐ALK fusion was shown to be variant 1 via comparative western blotting of the same variant harbored by the commonly used *ALK*‐rearranged lung cancer cell line NCI‐H3122 (Fig. 1D).

We next asked whether TR5 cancer cells carried unique genetic aberrations to explain their divergent phenotype. Panel sequencing of the TR3 and TR5 tumor tissues and their corresponding cultures, however, showed an identical set of somatic mutations (Fig. 1E and Table S2), suggesting that the difference between TR5 and other regions was phenotypic rather than mutational. The aggressive nature of the cancer was underscored by multiple mutations in tumor suppressor genes, including *TP53*, *CBFA2T3*, *PPP2R1A*, and *SMARCA4*. As expected, variant allele frequencies were substantially higher in cultures than in their respective tissues, indicating cancer cell enrichment in cultures. Overall, this demonstrates intratumor phenotypic heterogeneity and a genomic profile that underscores the aggressive nature of this *ALK*‐rearranged lung cancer sample.

### 3.2 Patient‐derived cells show moderate sensitivity to ALK inhibition

**Fig. 2:**
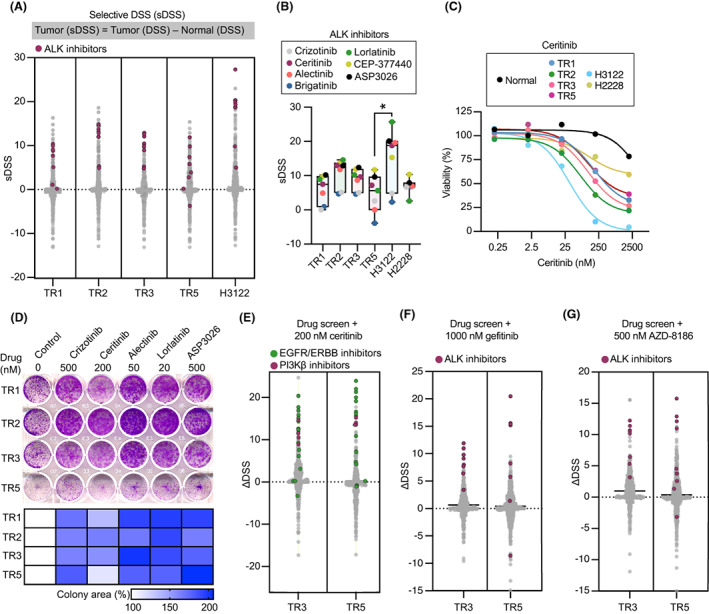
Pharmacological profiling reveals functional heterogeneity of an *ALK*‐rearranged NSCLC tumor. (A) Integrated results of drug sensitivity and resistance testing (DSRT) performed on tumor‐derived and H3122 cells; cancer‐selective hits were identified on the basis of selective drug sensitivity scores (DSSs). *ALK*‐rearranged lung cancer‐selective compounds targeting ALK are depicted as red (*n* = 1). (B) The selective DSSs of ALKi for indicated cell types. Each dot represents an individual drug and dots are color‐coded based on drug ID (*n* = 3). Whiskers represent minimum and maximum values. Wilcoxon matched‐pairs signed rank test *P* values are *P < 0.05. (C) Dose–response curves of normal lung or tumor‐derived cells, and H3122 or H2228 cells treated with the second‐generation ALKi, ceritinib (*n* = 3). (D) Representative images (top) of clonogenicity assays of tumor‐derived cultures treated for 15 days with IC_50_ concentrations of the indicated ALKi. IC_50_ concentrations were derived from a 3 day treatment experiment. Heat map (bottom) showing the quantification of change in colony with respect to vehicle control (*n* = 3). Integrated results of a combinatorial drug screen performed on cells derived from *ALK*‐rearranged tumor regions (TR3 and TR5), or from six normal lung tissues. Normal lung cells PLT26 were derived from healthy tissue neighboring the *ALK*‐rearranged tumor. DSRT screens were performed in combination with (E) 200 nm ceritinib (ALKi), (F) 1000 nm gefitinib (EGFRi), or (G) 500 nm AZD‐8186 (PI3Kβi) (*n* = 1).

To understand how the phenotypic heterogeneity of different tumor regions relates to functional differences, we undertook drug profiling of tumor‐derived cultures from four regions, and compared responses to those in patient‐matched normal lung tissue‐derived cells and *ALK*‐rearranged H3122 cells. This used a customized panel of 527 anticancer compounds representing both approved and investigational compounds, including > 100 compounds targeting ALK or its effector pathways (Table S3). Matching with the patient's clinical response, tumor‐derived cultures were mostly resistant to cisplatin and pemetrexed (Fig. S2A). The drug sensitivity score (DSS) was calculated for each drug response measurement by integrating the IC50 values, area under the curve (AUC), slope as well as top and lower asymptotes of each dose–response curve, allowing for rational comparisons across a large number of drugs and samples [19]. Selectivity was demonstrated by the finding that only *ALK*‐rearranged cancer cells, but not normal lung epithelial cells, showed sensitivity to ALK inhibitors (ALKi) (Fig. 2A–C and Fig. S2B,C). However, these ALKi responses were moderate when compared to those measured in *ALK*‐rearranged H3122 cells, but higher than in H2228 cells.

### 3.3 A combinatorial drug screen identifies PI3Kβ as a novel target to restore ALKi sensitivity

To assess long‐term sensitivity of tumor‐derived cultures to ALKi, cells were exposed to IC_50_ doses of five ALKi for 15 days. All tumor‐derived cultures showed paradoxical increased cell growth as evidenced by increased colony formation in presence of ALKi (Fig. 2D). We selected TR3 and TR5 cells for the remainder of the follow‐up experiments, as TR3 resembled the phenotypic features of TR1 and TR2, and TR5 uniquely differed from the other tumor‐derived cultures. Several ALK inhibitors were utilized in subsequent drug sensitivity experiments, with ceritinib being the most frequently used because it was the only second‐generation approved ALKi at the time of our investigation. With regard to the different sensitivity of tumor‐derived cultures to ALKi in short‐term and long‐term assays, we treated TR3 and TR5 cells with ceritinib and alectinib and measured the colonies every 3 days for up to 15 days to determine how long it would take for cancer cells to overcome the cytostatic effect of ALKi. Similar to the short‐term viability assay, the colony area of drug‐treated cells was lower than that of control cells at the 3‐day time point, while at later time points the colony areas of drug‐treated cells surpassed control cells. This suggested that 3–6 days are needed to overcome an ALKi‐induced halt in cell proliferation (Fig. S2C), eluding to loss of ALKi sensitivity in patient‐derived cells.

To identify treatments that may improve sensitivity to ALKi, we implemented a combinatorial drug screen on tumor‐derived cells using a drug panel in combination with 200 nm ceritinib. As expected, we identified combinatorial responses of ALKi plus EGFR inhibitors (EGFRi; *n* = 4) and pan‐ERBB inhibitors (*n* = 12). In addition, ALKi plus PI3Kβ inhibitors (PI3Kβi; *n* = 4) showed strong combinatorial responses (Fig. 2E and Fig. S3A–E). On the contrary, no combinatorial responses were seen between ALKi and PI3Kα isoform inhibitors or mTOR inhibitors (Fig. S3C). To cross‐validate our discoveries, we performed additional combinatorial screens in combination with gefitinib (an EGFRi) or AZD‐8186 (a PI3Kβ‐selective inhibitor). Both screens identified ALK as the most effective combination target (Fig. 2F,G and Fig. S3F), altogether demonstrating that partial sensitivity to ALKi primary tumor cells was regulated by EGFR and PI3Kβ and could be effectively overcome by combined inhibition of ALK and EGFR or PI3Kβ.

To understand the molecular mechanism for this limited ALKi sensitivity of tumor‐derived cultures, we analyzed signaling events following ceritinib treatment. Evaluation of phosphorylation cascades in TR3, TR5, and H3122 cells treated with different ceritinib concentrations showed a reduction in the level of ALK phosphorylation, and adaptive activation of EGFR and AKT was evident in all treated samples. When compared to TR3 and H3122, TR5 appeared to have higher levels of baseline ERK phosphorylation, while TR3 predominantly exhibited ERK rebound activation upon treatment with ceritinib (Fig. S4A,B). Next, to explore whether EGFR activation reduced sensitivity to ALKi, *ALK*‐rearranged lung cancer cells were cotreated with the EGFR ligand EGF and ceritinib. As expected, EGF treatment decreased the effect of ALKi on cell viability in all tested *ALK*‐rearranged lung cancer cell models (Fig. S4C). Since ALK inhibition led to adaptive activation of EGFR and downstream MAPK and PI3K‐AKT signaling, and as EGF perturbation dampened sensitivity to ALKi, the models evaluated in this study appear to exhibit EGFR‐driven ALKi resistance, matching findings of other studies [12, 22, 23].

### 3.4 Normal lung cells are insensitive to combined inhibition of ALK and PI3Kβ

**Fig. 3:**
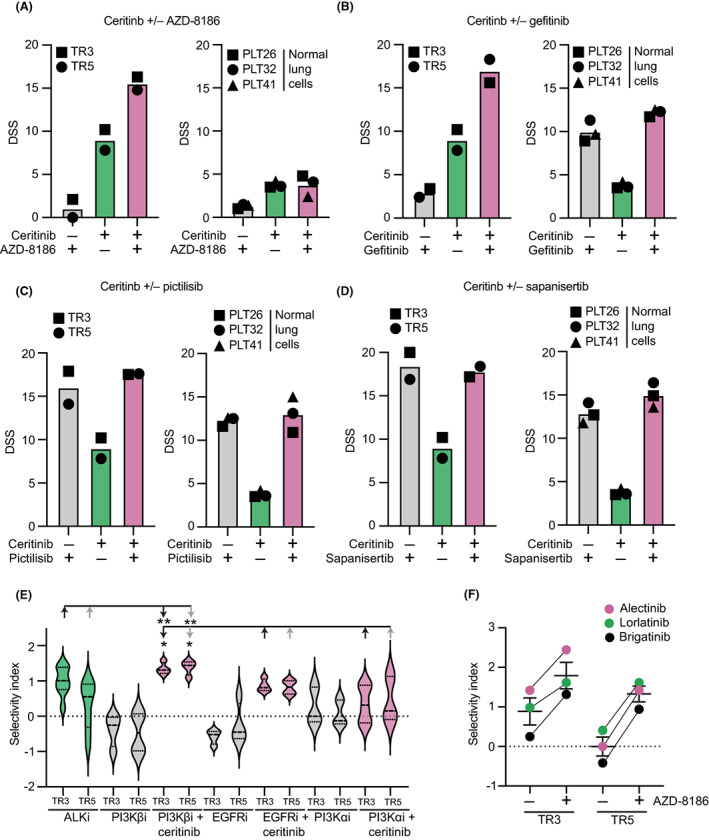
Implementation of combinatorial drug screen to identify sensitivities selective for *ALK*‐rearranged lung cancer. The drug sensitivity scores of ALKi as a single agent or in combination with (A) PI3Kβi; AZD‐8186, (B) EGFRi; gefitinib; (C) PI3Kαi; pictilisib or (D) mTORi; sapanisertib in *ALK*‐rearranged cancer cells and ALK wildtype normal lung cells (*n* = 2). (E) Selectivity index values p(IC_50_ *ALK*‐rearranged cells/IC_50_ ALK wildtype cells) for ALK inhibitors (*n* = 8) and its comparison with EGFR (*n* = 4), PI3Kβ (*n* = 4) and PI3Kα/mTOR inhibitors (*n* = 5) as a single agent or in combination with 200 nm ceritinib. (F) ALK inhibitor selectivity index values (*n* = 3) as a single agent or in combination with PI3Kβi; AZD‐8186. Error bars represent ± SEM. Student's *t* test *P* values are *P < 0.05, **P < 0.01.

To evaluate sensitivities and possible generic toxicities of compounds targeting the EGFR receptor family and the PI3K‐AKT–mTOR pathway, we utilized normal lung epithelial cells derived from three individuals. EGFR or pan‐ERBB inhibition produced toxic and antiproliferative responses in normal lung cells. Similarly, multiple compounds targeting various nodes of the PI3K‐AKT–mTOR pathway showed toxicities in the normal cells. However, compounds selectively targeting PI3Kβ, either on their own or in combinations with ALKi, did not detectably affect the normal lung epithelial cells (Fig. 3A–D and Fig. S5A–E). Furthermore, selectivity index analysis confirmed that the combination of ALKi and PI3Kβi provided a significantly wider therapeutic window in comparison to single ALKi or the combination of ALKi and EGFRi (Fig. 3E). In addition, selectivity index analysis demonstrated that next‐generation ALK inhibitors such as alectinib, brigatinib, and lorlatinib had superior selectivity when used in combination with PI3Kβi than when used alone (Fig. 3F). Therefore, the combination of PI3Kβi and ALKi acts highly selectively on *ALK*‐rearranged NSCLC cells, while the combination of EGFRi and ALKi appears to act non‐specifically, also targeting the normal epithelial cells.

### 3.5 Combined inhibition of ALK and PI3Kβ elicits synergistic responses in *ALK* ‐rearranged lung cancer cells

**Fig. 4:**
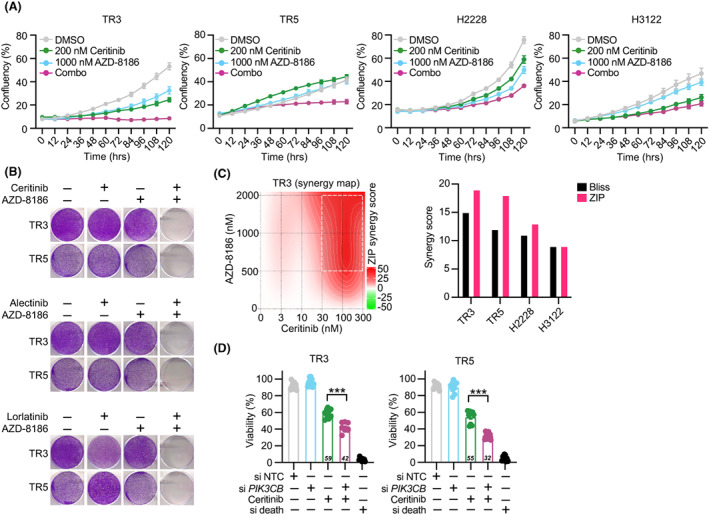
Targeting ALK in combination with PI3Kβ elicits ALK‐selective responses. (A) TR3, TR5, H2228, and H3122, cells were treated with vehicle (DMSO), 200 or 10 nm ceritinib, 1000 nm AZD‐8186 or the combination of ceritinib plus AZD‐8186; cell confluency was measured every 12 h for 5 days using Incucyte live cell imaging system (*n* = 3). (B) Representative images of clonogenicity assays of TR3 and TR5 cells treated for 15 days with ALKi or PI3Kβi or their combination for 15 days (*n* = 3). (C) Synergy map and synergy scores generated with SynergyFinder (https://synergyfinder.fimm.fi/) using clonogenicity assays quantified of TR3, TR5, H2228, and H3122 cells treated for 15 days with five different concentrations of ceritinib, three different concentrations of AZD‐8186, or their combination. (D) Percentage of viability of TR3 and TR5 cells when exposed to 200 nm ceritinib or vehicle in combination with siRNA‐mediated knockdown of *PIK3CB*, non‐targeting control (NTC). SiRNA and cell death siRNA were used as negative or positive controls, respectively. Viabilities of all samples were normalized to control untreated cells (*n* = 3). Error bars represent ± SEM. Student's *t* test *P* values are ***P < 0.001.

To widen the studies of the combinatorial effect of ALKi and PI3Kβi, drug response measurements were repeated and extended to two *ALK*‐rearranged lung cancer cell lines, H3122 and H2228. ALKi and PI3Kβi combination treatment effectively and synergistically prevented the proliferative and clonogenic potential of both the cell lines and the patient‐derived *ALK*‐rearranged cells (Fig. 4A–C). Using a wider concentration range of compounds and additionally by measuring death as a readout of drug responses we explored the synergistic potential of ALKi and PI3Kβi combination treatment. The previously detected viability‐based drug sensitivities were confirmed to be synergistic in TR3 and TR5 cells, but not in H3122 and H2228 cells (Fig. S6A–C). However, a cell death‐based readout showed a synergistic response of combinatorial ALK and PI3Kβ inhibition in H3122 cells (Fig. S6A–C). Furthermore, siRNA‐mediated gene silencing of *PIK3CB* in TR3 and TR5 mimicked the combinatorial responses seen with PI3Kβi (Fig. 4D), even though the knockdown of *PIK3CB* was only partial (Fig. S6D). The ALKi/PI3Kβi combination did not show an effect in four *KRAS* mutant lung cancer lines, validating the selectivity of this combination for *ALK*‐rearranged lung cancer (Fig. S6E).

Lastly, we assessed the *in vivo* efficacy of combinatorial ALKi and PI3Kβi treatment, and treated NSG mice (five animals per group) bearing subcutaneous tumors derived from TR3 and TR5 cells with ceritinib, AZD‐8186 or a combination of both treatments for 21 days. Both single agent and combination treatments were well‐tolerated with minor or no body weight loss over the course of treatment (Fig. S7A,B). Contrary to *in vitro* findings, both TR3 and TR5 cell‐derived tumors displayed strong sensitivity to ceritinib single therapy, and in both single and combination treatment arms, tumors became unmeasurable by the end of the treatment (Day 21; Fig. S7C,D). Within a week of treatment cessation, tumor growth resumed in both treatment arms (Fig. S7E,F), with tumor regrowth being notably slower in the combination treatment compared to the ceritinib arm (Fig. S7G,H).

### 3.6 Combinatorial ALKi plus PI3Kβi response appears independent of GPCR signaling and autophagy

Because autophagy and purinergic G‐protein‐coupled receptor (GPCR) activity have both been proposed to regulate ALKi sensitivity [23, 24, 25, 26], and that these processes are regulated by PI3Kβ, but not PI3Kα [24, 25], we investigated their involvement. Consistent with published data [26], ALK inhibition triggered autophagy, and the co‐targeting of ALK and PI3Kβ reduced autophagic vacuole formation (Fig. S8). We therefore tested whether autophagy inhibitors or autophagy and PI3Kα inhibitors could mimic the response to ALK plus PI3Kβ inhibition. However, none of the tested combinatorial treatments resulted in synergistic responses (Fig. S9A,B), suggesting that PI3Kβ acts beyond canonical PI3K‐AKT signaling and autophagy. Furthermore, while elevated P2Y subfamily of P2 purinergic GPCRs expression was reported to associate with ALKi resistance in both clinical samples and cultured cells [23], inhibition of P2YRs did not enhance ALKi response in our cell systems (Fig. S9C). In conclusion, our data suggest that PI3Kβ‐mediated inhibition of autophagy or P2YR signaling is not sufficient to induce the combinatorial effect with ALK inhibition in *ALK*‐rearranged cells.

### 3.7 Resistance to ALK inhibition is associated with MAPK and PI3K‐AKT pathway activation downstream of EGFR‐PI3Kβ

**Fig. 5:**
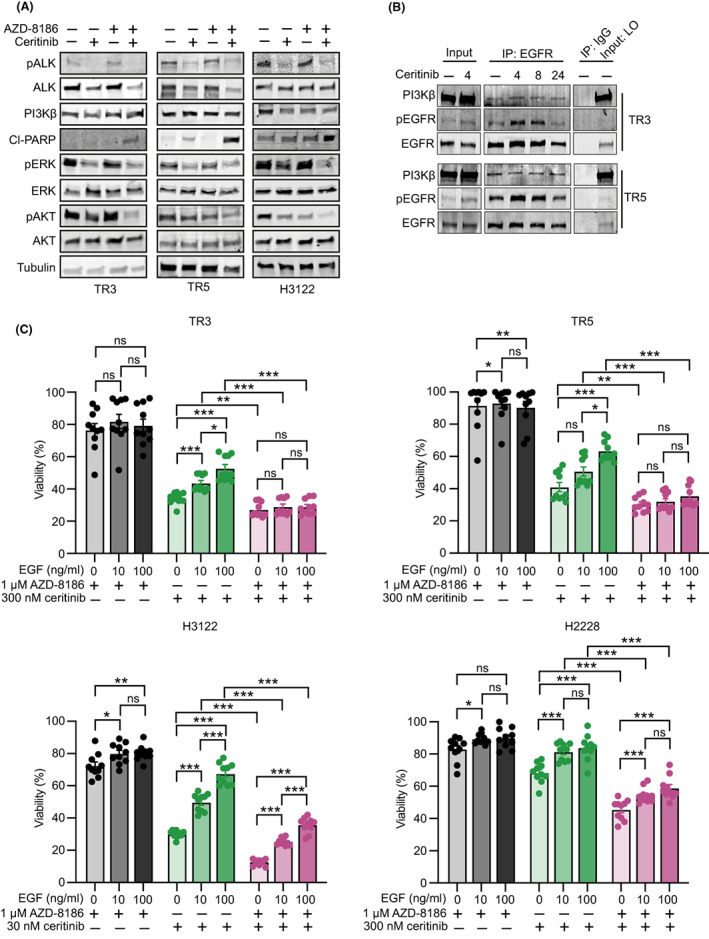
PI3Kβ contributes to cell growth and survival upon ALK inhibition in *ALK*‐rearranged lung cancer. (A) Immunoblots of TR3, TR5, and H3122 cells treated with vehicle control or 300 nm ceritinib (TR3/TR5) or 30 nm ceritinib (H3122) or 1000 nm AZD‐8186 or their combination for 72 h and probed with the indicated antibodies (*n* = 1). (B) TR3 and TR5 cells were treated with 300 nm ceritinib for different time points (4, 8, and 24 h) and lysates were immunoprecipitated with EGFR antibody. Precipitates were analyzed by immunoblotting and probed with the indicated antibodies (*n* = 2). (C) TR3, TR5, H3122, and H2228 cells were co‐treated with different doses of EGF (0, 10, and 100 ng·mL^−1^) and ceritinib or AZD‐8186 or their combination for 72 h. percentage viabilities of drug‐treated cells were normalized to cells co‐treated with different doses of EGF (0, 10, and 100 ng·mL^−1^) or DMSO (*n* = 3). Error bars represent ± SEM. Student's *t* test *P* values are *P < 0.05, **P < 0.01, ***P < 0.001. Ns, not significant.

To understand the consequences of combinatorial ALK plus PI3Kβ inhibition at the molecular level, we analyzed MAPK and PI3K‐AKT pathway activities in cells treated with vehicle, ceritinib, AZD‐8186, or their combination. In comparison with single treatments, cells treated with the combination showed a substantial reduction in both MAPK and PI3K‐AKT activities, as well as an increase in cell death evidenced by increased PARP cleavage accompanied by reduced ERK and AKT phosphorylation (Fig. 5A).

Next, we explored how PI3Kβ function is regulated upon ALK inhibition. Considering that the ERBB family member ERBB3 was reported to recruit PI3Kβ and thereby drive PI3Kα inhibitor resistance in *HER2*‐amplified and *PIK3CA* mutant cancers [27], we hypothesized that ALK inhibition may similarly activate EGFR and thereby regulate PI3Kβ function. We therefore immunoprecipitated EGFR from TR3 and TR5 cell lysates treated with DMSO control or 300 nm ceritinib for different times and assessed the phosphorylation of EGFR and PI3Kβ binding. EGFR phosphorylation was confirmed to significantly increase following ALK inhibition (Fig. 5B). Interestingly, PI3Kβ coprecipitated with EGFR, demonstrating that EGFR physically interacts with PI3Kβ in those cells (Fig. 5B). Importantly, treatment of TR3, TR5, H3122, or H2228 cells with EGF elicited increased resistance to ALK inhibition, while the combined inhibition of ALK and PI3Kβ rescued the EGF‐induced resistance, together suggesting that inhibition of PI3Kβ restricts EGFR‐mediated bypass resistance to ALK inhibition in *ALK*‐rearranged lung cancer (Fig. 5C and Fig. S10).

**Fig. 6:**
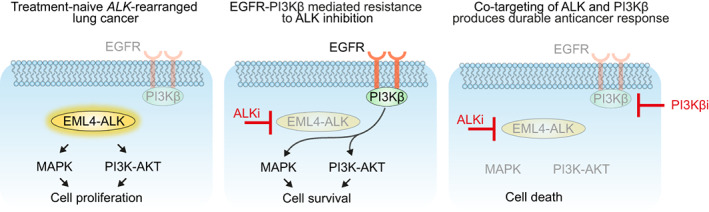
Graphical illustration describing resistance to ALKi in *ALK*‐rearranged lung cancers in which tumor cells are dependent on EGFR or PI3Kβ for cell growth and survival. Model for EML4‐ALK oncogene dependence in *ALK‐rearranged* lung cancer (left panel), in which tumor cells are primarily dependent on MAPK and PI3K‐AKT signaling. Inhibiting ALK (middle panel) results in an incomplete response and tumor cell survival due to MAPK and PI3K‐AKT pathway rebound activation. Our findings show that the EGFR‐PI3Kβ axis is responsible for this cytoprotective effect. In comparison to ALKi alone, combined inhibition of ALK and PI3Kβ (right panel) inhibits MAPK and PI3K‐AKT pathway rebound activation, promotes cell death, and induces a greater and more durable response.

In summary, dissection of the mechanisms underpinning combinatorial ALK and PI3Kβ inhibition efficacy suggest that the sensitization of *ALK*‐rearranged cancer cells to ALK inhibition by selective PI3Kβ inhibition was, at least in part, mediated through a blockade of EGFR‐mediated rebound activation of MAPK and PI3K‐AKT signaling, enhancing cell death (Fig. 6).

## 4 Discussion

ALK inhibition is an effective treatment option for *ALK*‐rearranged NSCLC. However, resistance to ALKi develops over time, and it is most often driven by ALK‐independent mechanisms [3]. We here studied primary cells derived from an aggressive *TP53* mutant and chemoresistant *ALK*‐rearranged tumor that exhibited region‐specific EMT features to identify novel/effective combination treatments to treat *ALK*‐rearranged NSCLC. We identified selective PI3Kβ inhibition as a promising novel target to potentiate ALK inhibition responses in *ALK*‐rearranged lung cancer. Importantly, this combinatorial effect was seen with all tested ALK inhibitors, including the most commonly used ALK inhibitor in the clinic, alectinib, and the third‐generation ALK inhibitor lorlatinib. Co‐targeting of ALK and PI3Kβ lead to blockade of both MAPK and PI3K‐AKT signaling, attenuated EGFR‐mediated adaptive resistance, and elicited tumor cell‐selective toxicity with efficacy even in *TP53* mutant *ALK*‐rearranged cells that had undergone EMT. Our study therefore demonstrates the utility of primary patient tumor‐derived cultures for pharmacogenomic profiling and dissection of drug resistance mechanisms.

Both the α and β PI3K class IA isoforms are ubiquitously expressed in both normal and malignant tissues, but these subunits are regulated differently, induce different modes of signaling, and have divergent roles in health and disease [28]. The PI3Kα isoform is a well‐established oncogenic driver that is frequently oncogenically mutated and constitutes a drug target for several cancer types, including lung cancer [28]. On the contrary, PI3Kβ has both kinase‐dependent and ‐independent functions as an effector of both RTKs and GPCRs [24]. Similar to PI3Kα, PI3Kβ participates in canonical PI3K‐AKT signaling, but also uniquely regulates DNA replication, cell cycle progression, nuclear envelope maintenance and nuclear pore complex assembly, autophagy, and apoptosis [24]. Despite PI3Kβ rarely being mutated in cancers, PI3Kβ expression correlates with poor prognosis in early‐stage NSCLC patients [29]. It has also been proposed as a drug target in PTEN‐deficient cancers [30] and in PI3Kα inhibitor‐resistant *PIK3CA* mutant cancers [27]. Our discovery of PI3Kβ as a target to enhance ALKi sensitivity in *ALK*‐rearranged lung cancer is unexpected, as we here found combinatorial ALKi/PI3Kβi sensitivity independent of *PTEN/PIK3CA* mutations or resistance to PI3Kα inhibitors. Despite lack of evidence for a clear role of autophagy or GPCR signaling in the PI3Kβ inhibitory responses identified here, PI3Kβ is a downstream node for signaling directed by RTKs that are widely implicated in driving resistance to ALKi, particularly IGF‐1R [31, 32], ERBB2/3 [23, 27] and GPCRs [23, 33]. The targeting of PI3Kβ may therefore counter a variety of bypass/acquired ALKi resistance mechanisms across sample types. Hence, our findings warrant further testing of the efficacy of PI3Kβi drug combinations on a larger set of patient‐derived samples. Due to the low success rate of establishing patient‐derived primary cultures, as underlined in our previous study [20], we could use cultures generated from different regions of a single tumor specimen. Our findings in the patient‐derived primary cultures were also confirmed in two commonly used *ALK*‐rearranged conventional lung cancer cell lines.

Previous research has identified various bypass signaling tracks that can mediate ALKi resistance, fostering the formulation of combinatorial treatment options that may overcome or even prevent drug resistance. The efficacy and tolerability of co‐targeting ALK with MEK (NCT03202940, NCT03087448) or mTORC1 (NCT02321501) are currently tested in patients with *ALK*‐rearranged NSCLC. In addition, following promising preclinical findings, the clinical testing of combinations co‐targeting ALK with AXL, SRC, KIT, MET or SHP2 have been proposed [3, 14]. Notably, the co‐inhibition of MEK, mTOR, AXL, SRC, KIT, MET or SHP2 did not enhance ALKi responses in our study. Eventually, only those combinations that exhibit both efficacy and manageable toxicity profiles will be viable treatment strategies. Use of organ‐matched healthy epithelial cells may help to exclude treatments that show generic toxicity [20]. For example, we detected sensitivity to HSP90 inhibition in both normal epithelial and cancer cell cultures, corroborating the finding that HSP90 inhibition induces toxicity without tumor‐selective response in patients carrying *ALK*‐rearranged NSCLC [34, 35]. Similarly, we found that even though EGFR inhibition potentiated the effect of ALK inhibition in the *ALK*‐rearranged lung cancer cells, normal lung epithelial cells also showed a generic toxic response to co‐targeting of EGFR, which aligns with severe toxicity in NSCLC patients receiving combinatorial treatments with ALKi and EGFRi [36, 37]. On the contrary, PI3Kβ inhibition, either on its own or combined with ALKi, did not detectably affect the normal lung epithelial cells. Our results suggest that EGFR and PI3Kβ share common pathways toward resistance, indicating that co‐targeting of ALK and PI3Kβ can be a safe strategy to counteract EGFR‐mediated adaptive resistance to ALKi. Promisingly, PI3Kβ‐selective inhibitors are being investigated in seven phase‐I/II clinical trials as a single agent or in combination with chemo‐ or immunotherapy (Table S4), following initial reports of satisfactory tolerability and efficacy [38, 39, 40].

From a heterogeneity standpoint, varied expression of lung adenocarcinoma markers, EMT, and tumor vasculature was observed across six different regions of the same tumor, suggesting that a single tumor biopsy would not provide a holistic assessment of this particular *ALK*‐rearranged cancer. Given that the ALKi plus PI3Kβi combination was effective on cells derived from *TP53* mutated tumor regions representing both epithelial and mesenchymal phenotypes, as well as established *ALK*‐rearranged H3122 (*TP53* mutant) and H2228 (*TP53* and *NFE2L2* mutant) cell lines, our findings suggest that this combination carries promise to counteract ALKi resistance associated with EMT, *TP53* or *NFE2L2* mutations [3, 41]. The *in vivo* treatment evaluation revealed that the combination treatment may provide a long‐term benefit, although the data included in the study lacked statistical significance and power of large cohorts. Therefore, to better evaluate the therapeutic potential of combination ALKi and PI3Kβi treatment, a sequential therapeutic approach and the use of a more resilient mouse strain, such as NMRI nu/nu, with a better tolerance for kinase inhibitors, may prove informative going ahead.

## 5 Conclusions

In summary, our study proposes PI3Kβ as a novel regulator of ALKi sensitivity in *ALK*‐rearranged lung cancer cells, showing efficacy even in aggressive *TP53* mutant and mesenchymal cells. Given that multiple salvage signals are activated upon resistance to ALKi, the co‐targeting of PI3Kβ provides a promising therapy option as PI3Kβ acts as a common effector of multiple salvage pathways, including EGFR, autophagy and GPCRs. Our data shows that this is the case for all clinically relevant ALK inhibitors. Importantly, we found that co‐targeting of PI3Kβ showed minimal toxicity in normal lung epithelial cells when compared with co‐targeting of EGFR, possibly providing for a safe clinical direction to treat *ALK*‐rearranged tumors.

## Conflict of interest

The authors declare no conflict of interest.

## Author contributions

SST, KW and EWV conceived and designed the study; SST performed *in vitro* experiments, analyzed the data, and generated the figures; MIM performed clinical pathology analyses; JS designed and performed the *in vivo* xenograft experiment; NL and AH implemented IHC staining and scanned slides; WS advised on the study design; SA characterized lung cancer cell lines; JR performed surgeries; JR, AK and MIM collected clinical data, received patients' informed consent, and managed the primary tissue workflow; MK performed genomic sequencing data analysis; SST, KW and EWV wrote the manuscript; MIM, JS, WS and ALL gave critical comments and corrections to the manuscript; EWV, JS, ALL and KW supervised the study.

### Peer review

The peer review history for this article is available at https://publons.com/publon/10.1002/1878‐0261.13342.

## Supporting information


**Fig. S1.** Tumor‐derived cultures retain genotypes and phenotypic features of tumors.
**Fig. S2.** *ALK*‐rearranged lung cancer cells exhibit a range of ALKi sensitivities.
**Fig. S3.** Effect of ALK and PI3Kβ inhibition is specific for tumor cells.
**Fig. S4.** Involvement of EGFR in dampening ALKi sensitivities.
**Fig. S5.** ALKi and PI3Kβi combination is cancer cell‐selective.
**Fig. S6.** PI3Kβi AZD‐8186 increases ceritinib efficacy in *ALK*‐rearranged lung cancer.
**Fig. S7.** *In vivo* testing of ceritinib, AZD‐8186 or their combination.
**Fig. S8.** ALK inhibition leads to autophagy.
**Fig. S9.** Inhibition of autophagy or P2Y receptors does not improve the response of ceritinib.
**Fig. S10.** Combined inhibition of ALK and PI3Kβ overcomes EGFR‐mediated resistance in *ALK*‐rearranged lung cancer cells.
**Table S1.** Details of primary antibodies used in immunohistochemistry and western blotting analyses.

**Table S2.** List of somatic mutations identified in tumor tissue and tumor‐derived cells.

**Table S3.** Drug library used for Drug Sensitivity and Resistance Testing.

**Table S4.** Clinical trial information for PI3Kβ inhibitors.

## Data Availability

The authors declare that the main data supporting the findings of this study are available within the article. Other data, such as IHC images midst sharable following GDPR regulations is available from the corresponding author upon request. Drug response curves, drug sensitivity scores, and IC50 values for all the tested drug or drug combinations will be made available upon request to the corresponding author.

## References

[1] Rikova K , Guo A , Zeng Q , Possemato A , Yu J , Haack H , et al. Global survey of phosphotyrosine signaling identifies oncogenic kinases in lung cancer. Cell. 2007;131:1190–203. 18083107 10.1016/j.cell.2007.11.025

[2] Soda M , Choi YL , Enomoto M , Takada S , Yamashita Y , Ishikawa S , et al. Identification of the transforming EML4‐ALK fusion gene in non‐small‐cell lung cancer. Nature. 2007;448:561–6. 17625570 10.1038/nature05945

[3] Lin JJ , Riely GJ , Shaw AT . Targeting ALK: precision medicine takes on drug resistance. Cancer Discov. 2017;7:137–55. 28122866 10.1158/2159-8290.CD-16-1123 PMC5296241

[4] Shaw AT , Hsu PP , Awad MM , Engelman JA . Tyrosine kinase gene rearrangements in epithelial malignancies. Nat Rev Cancer. 2013;13:772–87. 24132104 10.1038/nrc3612 PMC3902129

[5] Solomon BJ , Mok T , Kim DW , Wu YL , Nakagawa K , Mekhail T , et al. First‐line crizotinib versus chemotherapy in ALK‐positive lung cancer. N Engl J Med. 2014;371:2167–77. 25470694 10.1056/NEJMoa1408440

[6] Peters S , Camidge DR , Shaw AT , Gadgeel S , Ahn JS , Kim DW , et al. Alectinib versus Crizotinib in untreated ALK‐positive non‐small‐cell lung cancer. N Engl J Med. 2017;377:829–38. 28586279 10.1056/NEJMoa1704795

[7] Yoshida T , Oya Y , Tanaka K , Shimizu J , Horio Y , Kuroda H , et al. Differential Crizotinib response duration among ALK fusion variants in ALK‐positive non‐small‐cell lung cancer. J Clin Oncol. 2016;34:3383–9. 27354483 10.1200/JCO.2015.65.8732

[8] Aisner DL , Sholl LM , Berry LD , Rossi MR , Chen H , Fujimoto J , et al. The impact of smoking and TP53 mutations in lung adenocarcinoma patients with targetable mutations‐the lung cancer mutation consortium (LCMC2). Clin Cancer Res. 2018;24:1038–47. 29217530 10.1158/1078-0432.CCR-17-2289 PMC7008001

[9] Kron A , Alidousty C , Scheffler M , Merkelbach‐Bruse S , Seidel D , Riedel R , et al. Impact of TP53 mutation status on systemic treatment outcome in ALK‐rearranged non‐small‐cell lung cancer. Ann Oncol. 2018;29:2068–75. 30165392 10.1093/annonc/mdy333 PMC6225899

[10] Qin K , Hou H , Liang Y , Zhang X . Prognostic value of TP53 concurrent mutations for EGFR‐ TKIs and ALK‐TKIs based targeted therapy in advanced non‐small cell lung cancer: a meta‐analysis. BMC Cancer. 2020;20:328. 32299384 10.1186/s12885-020-06805-5 PMC7164297

[11] Doebele RC , Pilling AB , Aisner DL , Kutateladze TG , Le AT , Weickhardt AJ , et al. Mechanisms of resistance to crizotinib in patients with ALK gene rearranged non‐small cell lung cancer. Clin Cancer Res. 2012;18:1472–82. 22235099 10.1158/1078-0432.CCR-11-2906 PMC3311875

[12] Katayama R , Shaw AT , Khan TM , Mino‐Kenudson M , Solomon BJ , Halmos B , et al. Mechanisms of acquired crizotinib resistance in ALK‐rearranged lung cancers. Sci Transl Med. 2012;4:120ra117. 10.1126/scitranslmed.3003316 PMC3385512 22277784

[13] Crystal AS , Shaw AT , Sequist LV , Friboulet L , Niederst MJ , Lockerman EL , et al. Patient‐derived models of acquired resistance can identify effective drug combinations for cancer. Science. 2014;346:1480–6. 25394791 10.1126/science.1254721 PMC4388482

[14] Dardaei L , Wang HQ , Singh M , Fordjour P , Shaw KX , Yoda S , et al. SHP2 inhibition restores sensitivity in ALK‐rearranged non‐small‐cell lung cancer resistant to ALK inhibitors. Nat Med. 2018;24:512–7. 29505033 10.1038/nm.4497 PMC6343825

[15] Tsuji T , Ozasa H , Aoki W , Aburaya S , Yamamoto Funazo T , Furugaki K , et al. YAP1 mediates survival of ALK‐rearranged lung cancer cells treated with alectinib via pro‐apoptotic protein regulation. Nat Commun. 2020;11:74. 10.1038/s41467-019-13771-5 31900393 PMC6941996

[16] Liu X , Krawczyk E , Suprynowicz FA , Palechor‐Ceron N , Yuan H , Dakic A , et al. Conditional reprogramming and long‐term expansion of normal and tumor cells from human biospecimens. Nat Protoc. 2017;12:439–51. 28125105 10.1038/nprot.2016.174 PMC6195120

[17] Talwelkar SS , Nagaraj AS , Devlin JR , Hemmes A , Potdar S , Kiss EA , et al. Receptor tyrosine kinase signaling networks define sensitivity to ERBB inhibition and stratify Kras‐mutant lung cancers. Mol Cancer Ther. 2019;18:1863–74. 31320402 10.1158/1535-7163.MCT-18-0573

[18] Potdar S , Ianevski A , Mpindi JP , Bychkov D , Fiere C , Ianevski P , et al. Breeze: an integrated quality control and data analysis application for high‐throughput drug screening. Bioinformatics. 2020;36:3602–4. 32119072 10.1093/bioinformatics/btaa138 PMC7267830

[19] Yadav B , Pemovska T , Szwajda A , Kulesskiy E , Kontro M , Karjalainen R , et al. Quantitative scoring of differential drug sensitivity for individually optimized anticancer therapies. Sci Rep. 2014;4:5193. 10.1038/srep05193 24898935 PMC4046135

[20] Talwelkar SS , Mayranpaa MI , Soraas L , Potdar S , Bao J , Hemmes A , et al. Functional diagnostics using fresh uncultured lung tumor cells to guide personalized treatments. Cell Rep Med. 2021;2:100373. 10.1016/j.xcrm.2021.100373 34467250 PMC8385325

[21] Dufva O , Kankainen M , Kelkka T , Sekiguchi N , Awad SA , Eldfors S , et al. Aggressive natural killer‐cell leukemia mutational landscape and drug profiling highlight JAK‐STAT signaling as therapeutic target. Nat Commun. 2018;9:1567. 10.1038/s41467-018-03987-2 29674644 PMC5908809

[22] Sasaki T , Koivunen J , Ogino A , Yanagita M , Nikiforow S , Zheng W , et al. A novel ALK secondary mutation and EGFR signaling cause resistance to ALK kinase inhibitors. Cancer Res. 2011;71:6051–60. 21791641 10.1158/0008-5472.CAN-11-1340 PMC3278914

[23] Wilson FH , Johannessen CM , Piccioni F , Tamayo P , Kim JW , Van Allen EM , et al. A functional landscape of resistance to ALK inhibition in lung cancer. Cancer Cell. 2015;27:397–408. 25759024 10.1016/j.ccell.2015.02.005 PMC4398996

[24] Bresnick AR , Backer JM . PI3Kbeta‐a versatile transducer for GPCR, RTK, and small GTPase signaling. Endocrinology. 2019;160:536–55. 30601996 10.1210/en.2018-00843 PMC6375709

[25] Dou Z , Pan JA , Dbouk HA , Ballou LM , DeLeon JL , Fan Y , et al. Class IA PI3K p110beta subunit promotes autophagy through Rab5 small GTPase in response to growth factor limitation. Mol Cell. 2013;50:29–42. 23434372 10.1016/j.molcel.2013.01.022 PMC3628298

[26] Frentzel J , Sorrentino D , Giuriato S . Targeting autophagy in ALK‐associated cancers. Cancers (Basel). 2017;9:161. 10.3390/cancers9120161 29186933 PMC5742809

[27] Costa C , Ebi H , Martini M , Beausoleil SA , Faber AC , Jakubik CT , et al. Measurement of PIP3 levels reveals an unexpected role for p110beta in early adaptive responses to p110alpha‐specific inhibitors in luminal breast cancer. Cancer Cell. 2015;27:97–108. 25544637 10.1016/j.ccell.2014.11.007 PMC4745884

[28] Thorpe LM , Yuzugullu H , Zhao JJ . PI3K in cancer: divergent roles of isoforms, modes of activation and therapeutic targeting. Nat Rev Cancer. 2015;15:7–24. 25533673 10.1038/nrc3860 PMC4384662

[29] Lee JS , Lee HW , Lee EH , Park MI , Lee JS , Kim MS , et al. Prognostic significance of phosphoinositide 3‐kinase p110alpha and p110beta isoforms in non‐small cell lung cancer. Int J Clin Exp Pathol. 2018;11:1554–61. 31938253 PMC6958152

[30] Hancox U , Cosulich S , Hanson L , Trigwell C , Lenaghan C , Ellston R , et al. Inhibition of PI3Kbeta signaling with AZD8186 inhibits growth of PTEN‐deficient breast and prostate tumors alone and in combination with docetaxel. Mol Cancer Ther. 2015;14:48–58. 25398829 10.1158/1535-7163.MCT-14-0406

[31] Leroy C , Ramos P , Cornille K , Bonenfant D , Fritsch C , Voshol H , et al. Activation of IGF1R/p110beta/AKT/mTOR confers resistance to alpha‐specific PI3K inhibition. Breast Cancer Res. 2016;18:41. 10.1186/s13058-016-0697-1 27048245 PMC4820873

[32] Lovly CM , McDonald NT , Chen H , Ortiz‐Cuaran S , Heukamp LC , Yan Y , et al. Rationale for co‐targeting IGF‐1R and ALK in ALK fusion‐positive lung cancer. Nat Med. 2014;20:1027–34. 25173427 10.1038/nm.3667 PMC4159407

[33] Dbouk HA , Vadas O , Shymanets A , Burke JE , Salamon RS , Khalil BD , et al. G protein‐coupled receptor‐mediated activation of p110beta by Gbetagamma is required for cellular transformation and invasiveness. Sci Signal. 2012;5:ra89. 10.1126/scisignal.2003264 23211529 PMC3979326

[34] Pillai RN , Ramalingam SS . Throwing more cold water on heat shock protein 90 inhibitors in NSCLC. J Thorac Oncol. 2018;13:473–4. 29576286 10.1016/j.jtho.2018.02.010

[35] Sang J , Acquaviva J , Friedland JC , Smith DL , Sequeira M , Zhang C , et al. Targeted inhibition of the molecular chaperone Hsp90 overcomes ALK inhibitor resistance in non‐small cell lung cancer. Cancer Discov. 2013;3:430–43. 23533265 10.1158/2159-8290.CD-12-0440 PMC4086149

[36] Janne PA , Shaw AT , Camidge DR , Giaccone G , Shreeve SM , Tang Y , et al. Combined Pan‐HER and ALK/ROS1/MET inhibition with Dacomitinib and Crizotinib in advanced non‐small cell lung cancer: results of a phase I study. J Thorac Oncol. 2016;11:737–47. 26899759 10.1016/j.jtho.2016.01.022

[37] Yang Z , Tam KY . Combination strategies using EGFR‐TKi in NSCLC therapy: learning from the gap between pre‐clinical results and clinical outcomes. Int J Biol Sci. 2018;14:204–16. 29483838 10.7150/ijbs.22955 PMC5821041

[38] Choudhury AD , Higano CS , de Bono JS , Cook N , Rathkopf DE , Wisinski KB , et al. A phase I study investigating AZD8186, a potent and selective inhibitor of PI3Kbeta/delta, in patients with advanced solid tumors. Clin Cancer Res. 2022;28:2257–69. 35247924 10.1158/1078-0432.CCR-21-3087 PMC9662946

[39] Hansen AR , Shapiro G , Do KT , Kumar R , Martin‐Liberal J , Higano CS , et al. A first in human phase I study of AZD8186, a potent and selective inhibitor of PI3K in patients with advanced solid tumours as monotherapy and in combination with the dual mTORC1/2 inhibitor vistusertib (AZD2014) or abiraterone acetate. J Clin Oncol. 2017;35:2570. 10.1200/JCO.2017.35.15_suppl.2570

[40] Janku F , Yap TA , Meric‐Bernstam F . Targeting the PI3K pathway in cancer: are we making headway? Nat Rev Clin Oncol. 2018;15:273–91. 29508857 10.1038/nrclinonc.2018.28

[41] Krall EB , Wang B , Munoz DM , Ilic N , Raghavan S , Niederst MJ , et al. KEAP1 loss modulates sensitivity to kinase targeted therapy in lung cancer. Elife. 2017;6:e18970. 10.7554/eLife.18970 28145866 PMC5305212

