# Supplementary material for: PI3Kβ inhibition enhances ALK‐inhibitor sensitivity in ALK ‐rearranged lung cancer
Source: Mol Oncol. 2023 Jan 25;17(5):747–64. doi: 10.1002/1878-0261.13342 (PMC10158778; doi:10.1002/1878-0261.13342)
Supplement: Supplementary file 1 — Fig. S1. Tumor‐derived cultures retain genotypes and phenotypic features of tumors. Fig. S2. ALK‐rearranged lung cancer cells exhibit a range of ALKi sensitivities. Fig. S3. Effect of ALK and PI3Kβ inhibition is specific for tumor cells. Fig. S4. Involvement of EGFR in dampening ALKi sensitivities. Fig. S5. ALKi and PI3Kβi combination is cancer cell‐selective. Fig. S6. PI3Kβi AZD‐8186 increases ceritinib efficacy in ALK‐rearranged lung cancer. Fig. S7. In vivo testing of ceritinib, AZD‐8186 or their combination. Fig. S8. ALK inhibition leads to autophagy. Fig. S9. Inhibition of autophagy or P2Y receptors does not improve the response of ceritinib. Fig. S10. Combined inhibition of ALK and PI3Kβ overcomes EGFR‐mediated resistance in ALK‐rearranged lung cancer cells. Table S1. Details of primary antibodies used in immunohistochemistry and western blotting analyses. [file MOL2-17-747-s004.docx]

### SUPPORTING INFORMATION

**Table of contents:**

**Supplementary Figures/Tables and Figure/Table Legends**

- **Figure S1.** Tumor-derived cultures retain genotypes and phenotypic features of tumors

● **Figure S2.** *ALK*-rearranged lung cancer cells exhibit a range of ALKi sensitivities

● **Figure S3.** The effect of ALK and PI3Kβ inhibition is specific for tumor cells

● **Figure S4.** Involvement of EGFR in dampening ALKi sensitivities

● **Figure S5.** The ALKi and PI3Kβi combination is cancer cell-selective

- **Figure S6.** The PI3Kβi AZD-8186 increases ceritinib efficacy in *ALK*-rearranged lung cancer
- **Figure S7.** *In vivo* testing of ceritinib, AZD-8186 or their combination
- **Figure S8.** ALK inhibition leads to autophagy
- **Figure S9.** Inhibition of autophagy or P2Y receptors does not improve the response of ceritinib
- **Figure S10.** Combined inhibition of ALK and PI3Kβ overcomes EGFR-mediated resistance in *ALK*-rearranged lung cancer cells
- Table S1. Details of primary antibodies used in immunohistochemistry and western blotting analyses
- **Table S2.** List of somatic mutations identified in tumor tissue and tumor-derived cells
- **Table S3.** Drug library used for Drug Sensitivity and Resistance Testing
- **Table S4.** Clinical trial information for PI3Kβ inhibitors


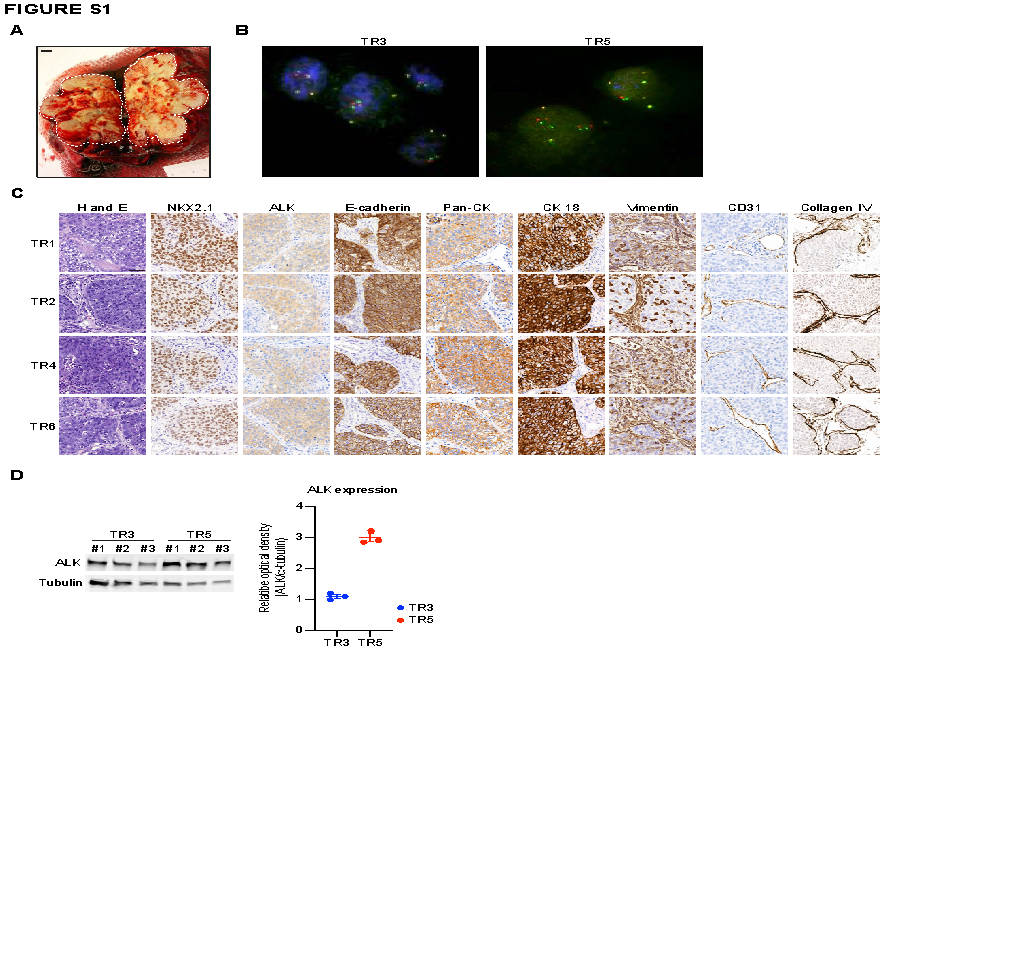


**Figure S1. Tumor-derived cultures retain genotypes and phenotypic features of tumors.** (A) Image of the surgically removed tumor tissue used to collect samples from multiple regions. The scale bar corresponds to one cm. (B) FISH analysis of TR3- and TR5-derived cells for ALK rearrangement. (C) Extended dataset for Figure 1B. Representative images of hematoxylin and eosin (H&E) and IHC staining performed on different regions of the *ALK*-rearranged tumor tissue. The scale bar corresponds to 50 μm. (D) Immunoblots (left) of *ALK*-rearranged tumor-derived cultures and probed with the indicated antibodies. Quantification (right) of relative expression of ALK in TR3 and TR5 cells. Error bars represent ± SEM.

**
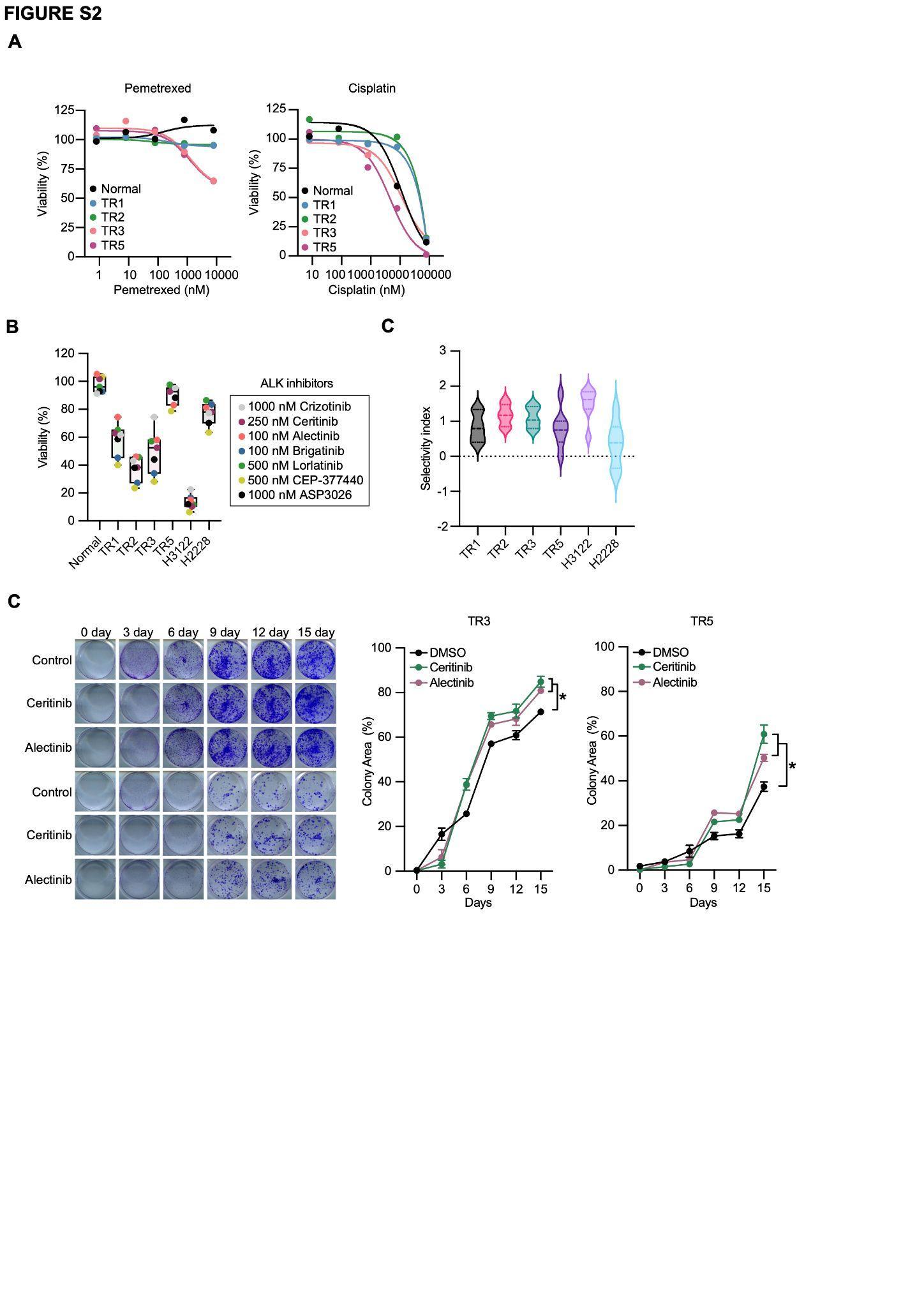
**

**Figure S2. *ALK*-rearranged lung cancer cells exhibit a range of ALKi sensitivities.** (A) Dose-response curves of normal lung or tumor-derived cells treated with pemetrexed and cisplatin; these were chemotherapy drugs given to the patient. (B) Percentage viabilities of cells treated with ALK inhibitors; each dot in box chart represent the viability of different ALK inhibitors. Whiskers represent minimum and maximum values. (C) Selectivity index values p(IC_50_ *ALK*-rearranged cells/IC_50_ ALK wildtype cells) for ALK inhibitors (n=7). Representative images (left) of clonogenicity assays of tumor-derived cultures treated for 0, 3, 6, 9, 12, and 15 days with IC50 concentrations of indicated ALKi. IC50 concentrations were derived from a three day treatment experiment. Graphs (right) showing the quantification of change in colony are with respect to 0 day of respective sample. Error bars represent ± SEM. Student’s t test p values are * p < 0.05.


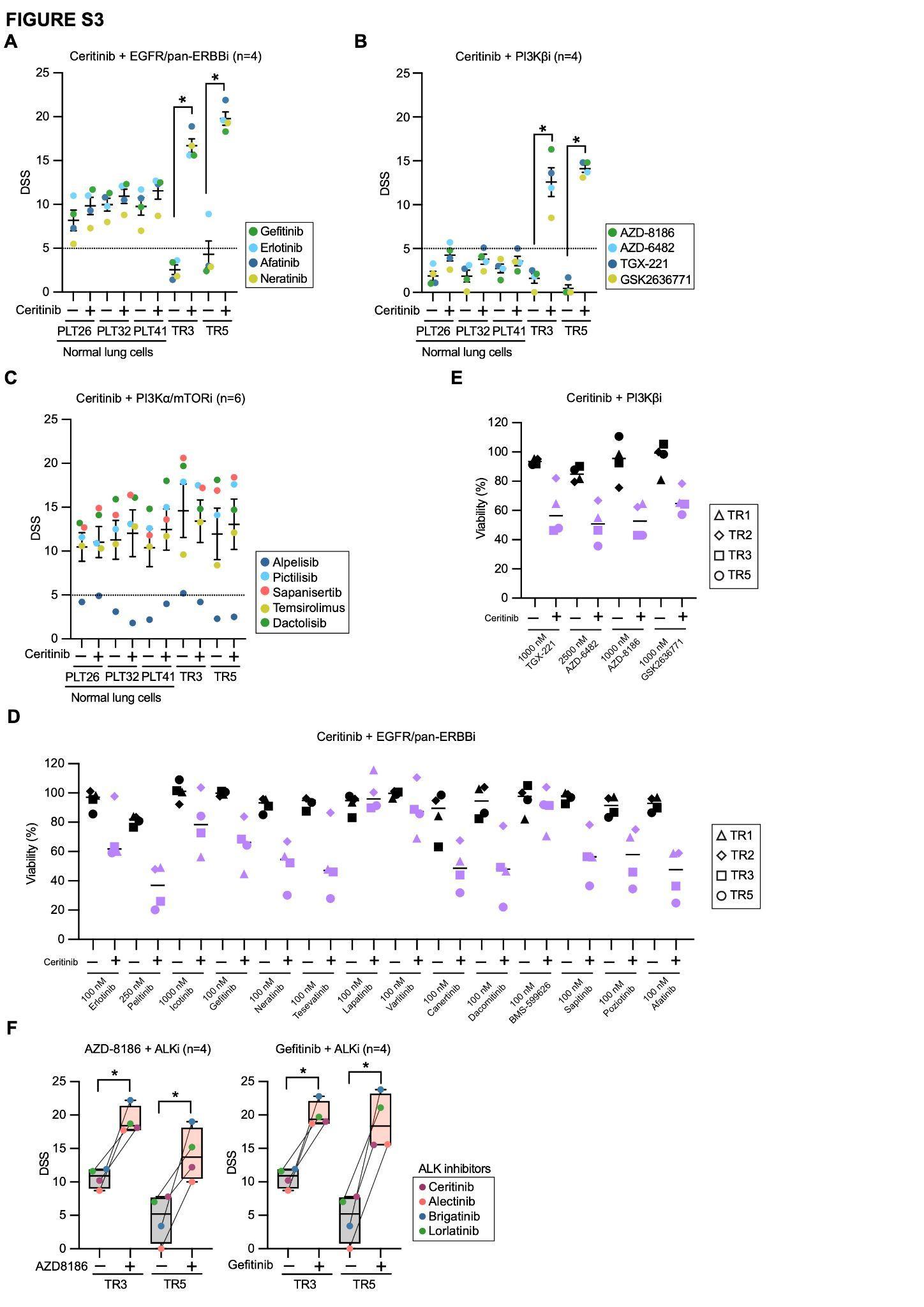


**Figure S3. The effect of ALK and PI3Kβ inhibition is specific for tumor cells.** Normal lung or tumor-derived cells were treated with combination of (A) EGFRi or (B) PI3Kβi or (C) PI3Kα/mTOR inhibitors, each in the absence (-) and presence (+) of 200 nM ceritinib; each dot in box chart represent the DSSs of single agents or combinations. Error bars represent standard error of mean. Percentage viabilities of cells treated with combination of (D) EGFRi or (E) PI3Kβi, each in the absence (-) and presence (+) of 200 nM ceritinib; each dot in box chart represent the viability of of single agents or combinations. (F) The DSSs of ALKi as a single agent or in combination with 500 nM AZD-8186 or 1000 nM gefitinib. Error bars represent minimum and maximum values. Student’s *t* test *p* values are * p < 0.05.


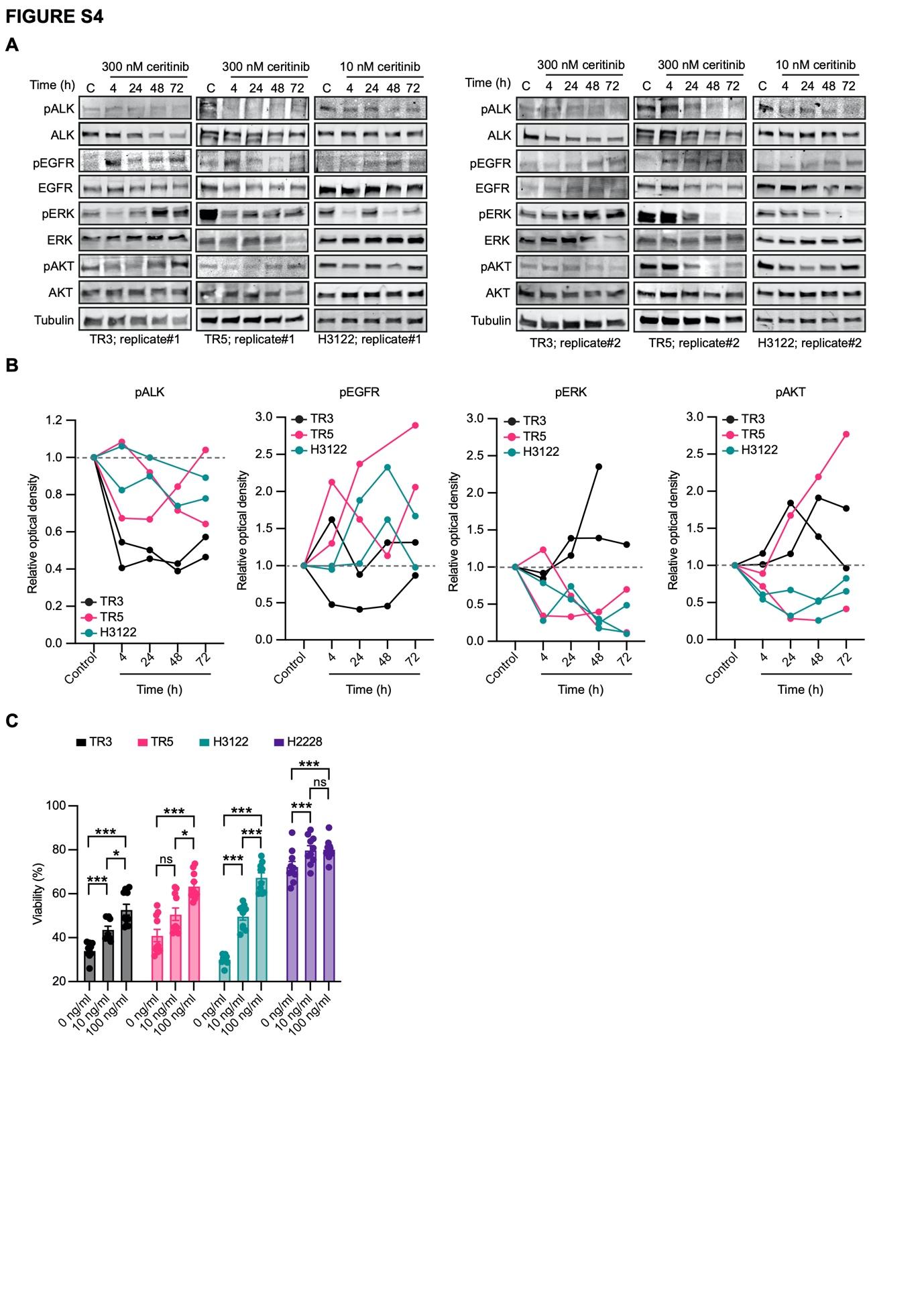


**Figure S4. Involvement of EGFR in dampening ALKi sensitivities.** (A) Immunoblots of TR3, TR5, and H3122 cells treated with vehicle (C; DMSO) and or treated with 300 nM ceritinib (TR3/TR5) or 10 nM ceritinib (H3122) for various time points (4, 24, 48, and 72 h) and probed with the indicated antibodies. ALK and phospho-ALK bands indicate variant 1 of EML4-ALK protein (117 kDa). (B) Relative expression level of phospho proteins presented in (A). (C) TR3, TR5, H3122, and H2228 cells were co-treated with different doses of EGF (0, 10, and 100 ng/ml) and ceritinib for 72 h. Percentage viabilities of drug-treated cells were normalized to cells co-treated with different doses of EGF (0, 10, and 100 ng/ml) or DMSO. Error bars represent ± SEM. Student’s t test p values are * p < 0.05, *** p <0.001. Abbreviation: ns: not significant.


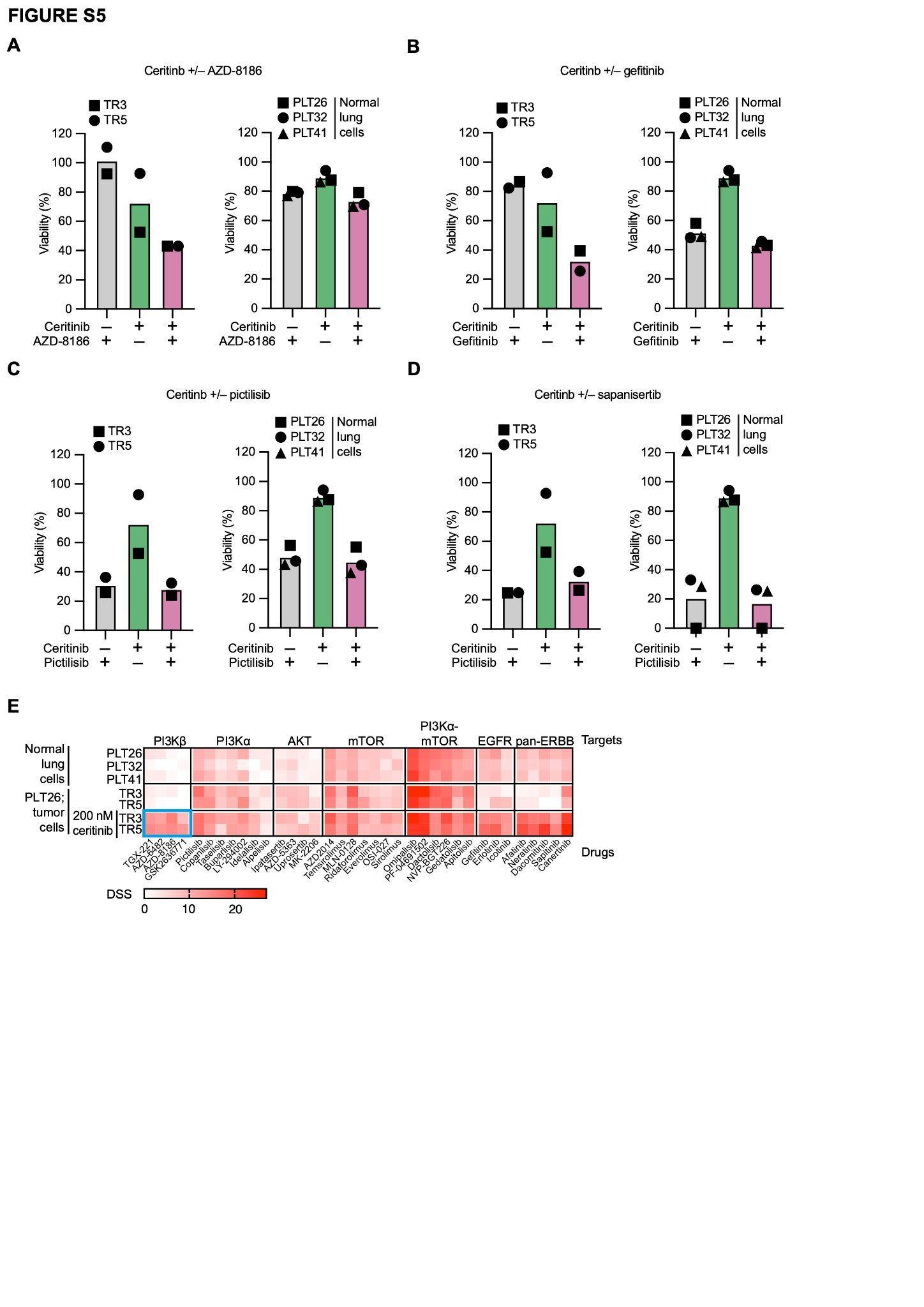


**Figure S5. The ALKi and PI3Kβi combination is cancer cell-selective.** The percentage viabilities of ALKi as a single agent or in combination with (A) PI3Kβi; AZD-8186, (B) EGFRi; gefitinib; (C) PI3Kαi; pictilisib or (D) mTORi; sapanisertib in *ALK*-rearranged cancer cells and ALK wildtype normal lung cells. (E) Heatmap showing the drug sensitivity scores of compounds inhibiting the indicated targets.

**
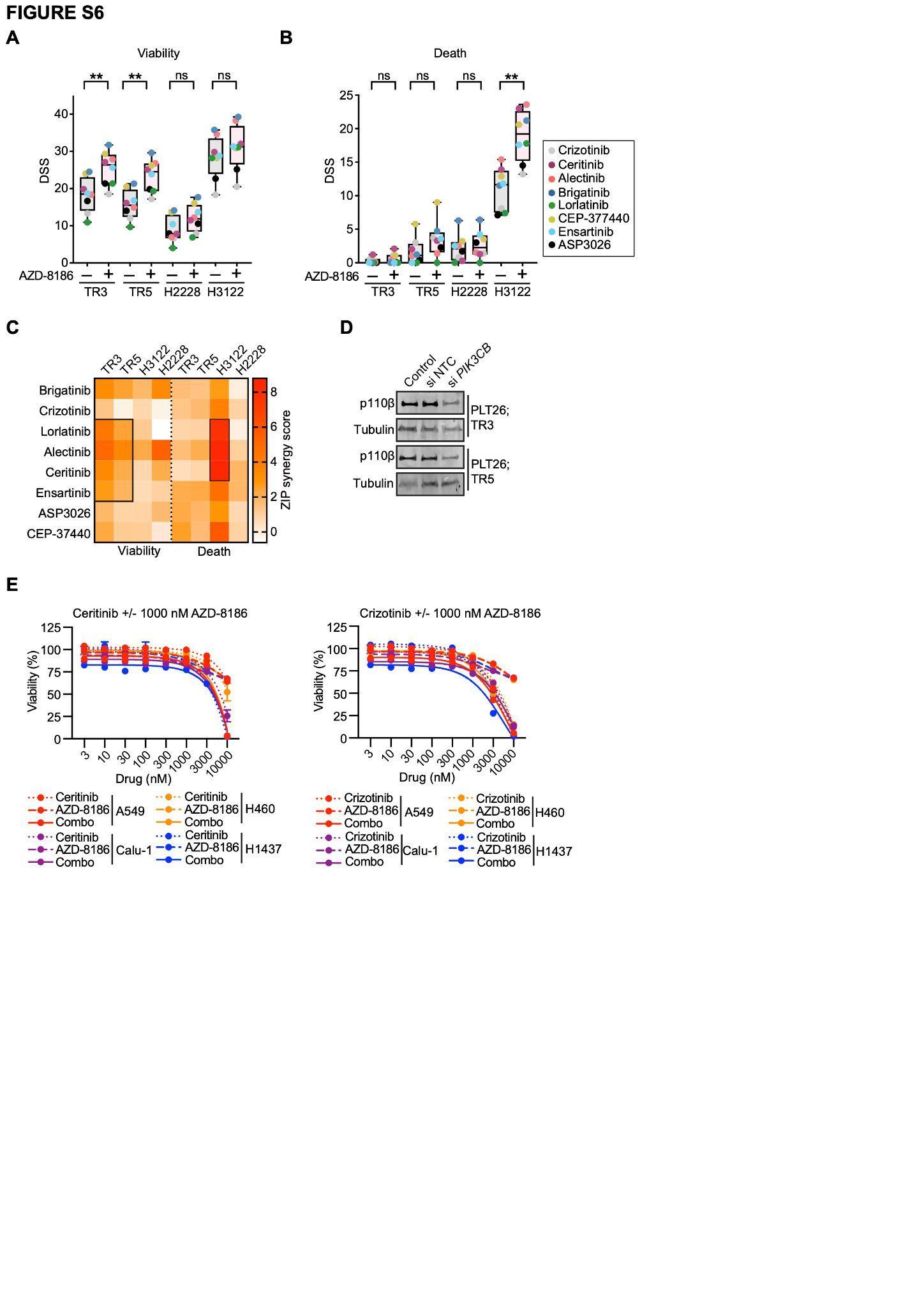
**

**Figure S6. The PI3Kβi AZD-8186 increases ceritinib efficacy in *ALK*-rearranged lung cancer.** The DSSs of single ALKi treatment (nine doses between 0.5 and 5000 nM) or combination treatment with ALKi plus 500 nM AZD-8186. Drug sensitivities were either measured by (A) a CellTiter-Glo-based cell viability assay or (B) a CellTox Green-based cell death assay. Whiskers represent minimum and maximum values. (C) Heatmap representing zero interaction potency (ZIP)-based synergy scores calculated for each combination of ALKi plus 500 nM AZD-8186. (D) Immunoblots of TR3 and TR5 cells transfected with NTC or *PIK3CB* siRNA and probed with the indicated antibodies. (E) *ALK* wildtype and *KRAS* mutant lung cancer cells (n=4) were treated with ceritinib, crizotinib, AZD-8186, or the combination of ceritinib or crizotinib plus 1000 nM AZD-8186. Error bars represent ± SEM. Student’s t test p values are ** p < 0.01. Abbreviation: ns: not significant.


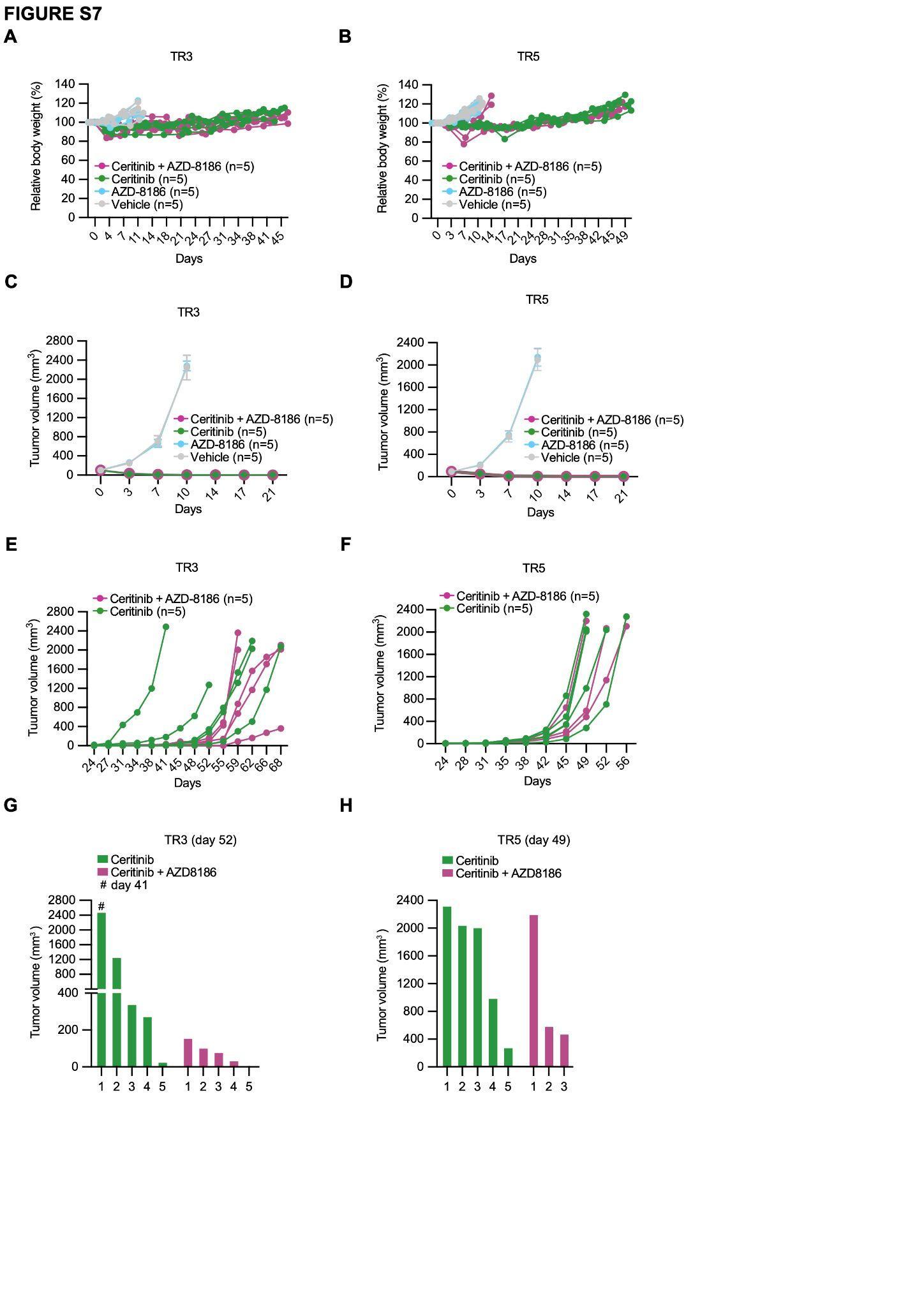


**Figure S7. *In vivo* testing of ceritinib, AZD-8186 or their combination.** Mice (n=5 per treatment arm) harboring TR3 and TR5 tumors were administered with vehicle, 25 mg/kg/day ceritinib, 2$\times$25 mg/kg/day AZD-8186, or the combination of ceritinib and AZD-8186, for 21 days. Body weights of mice bearing subcutaneous (A) TR3 and (B) TR5 tumors. Graph shows the change in tumor volume (mm3) of (C) TR3 and (D) TR5 during the treatment span. Graph shows the change in tumor volume (mm3) of (E) TR3 and (F) TR5 during the post treatment withdrawal. Waterfall plots for individual mice treated with the indicated treatments show the tumor volume (mm3) of (G) TR3 at day 52 and (H) TR5 at day 49 post-treatment withdrawal. The mouse in the TR3 ceritinib treatment arm, shown by # in the waterfall plot, was euthanized on day 41 because the tumor volume had reached its maximum permissible limit. The waterfall plot for TR5 shows three mice for the combination therapy arm since two mice were euthanized on day 15 due to a distended abdomen. Error bars represent ± SEM.


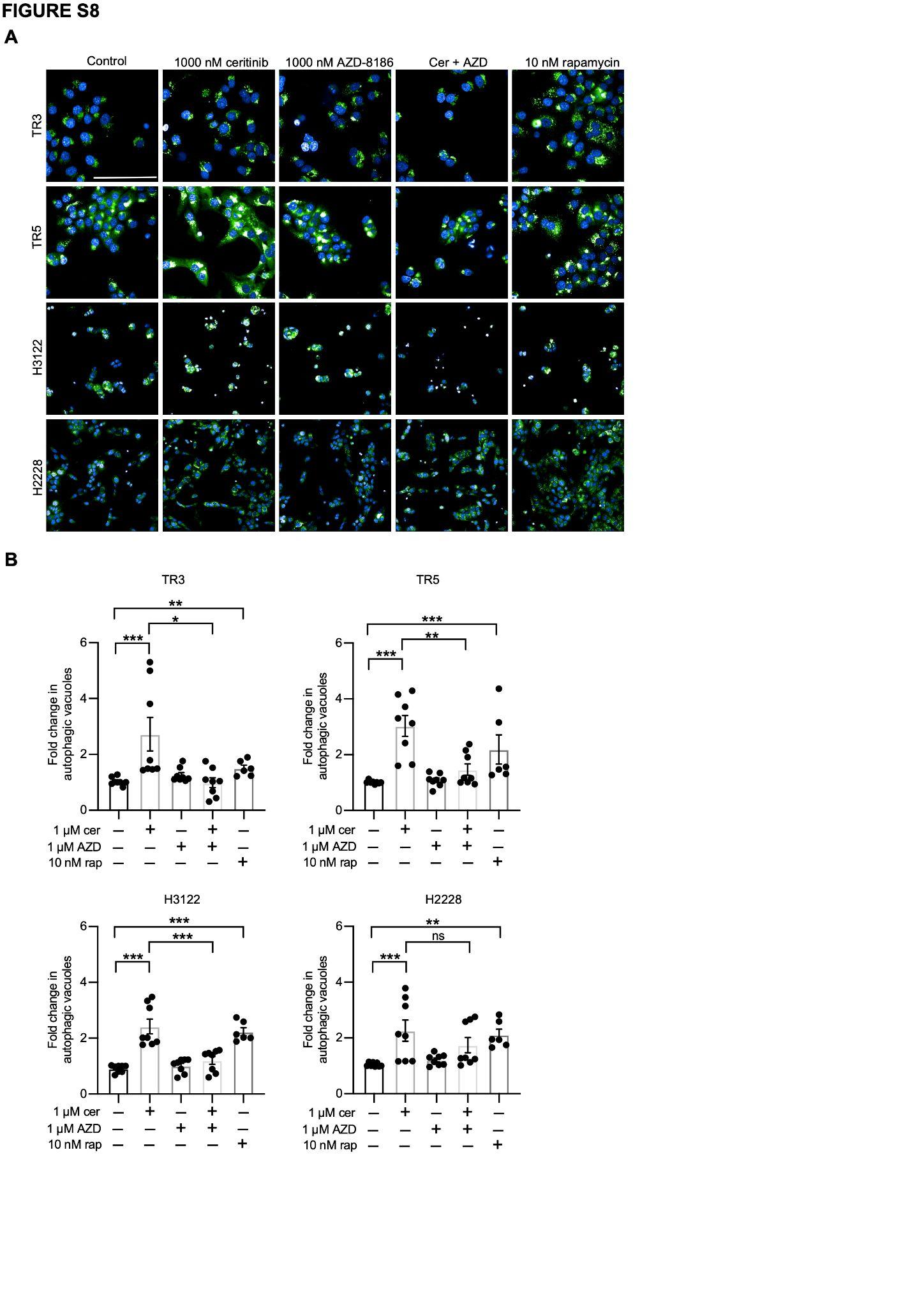


**Figure S8. ALK inhibition leads to autophagy.** (A) Representative images of TR5 and TR3 cells treated with vehicle control, 1000 nM ceritinib (cer), 1000 nM AZD-8186 (AZD), or their combination for 24 h. Before imaging, cells were stained with the Cyto-ID Green Detection Reagent to visualize autophagic vacuoles. Cells treated with 10 nM rapamycin (rap) served as a positive control. The scale bar corresponds to 100 μm. (B) Bar graph representing fold changes in the number of autophagic vacuoles in drug-treated cells relative to vehicle control. Error bars represent ± SEM. Student’s *t* test *p* values are * p < 0.05, ** p <0.01, *** p <0.001. Abbreviation: ns: not significant.

**
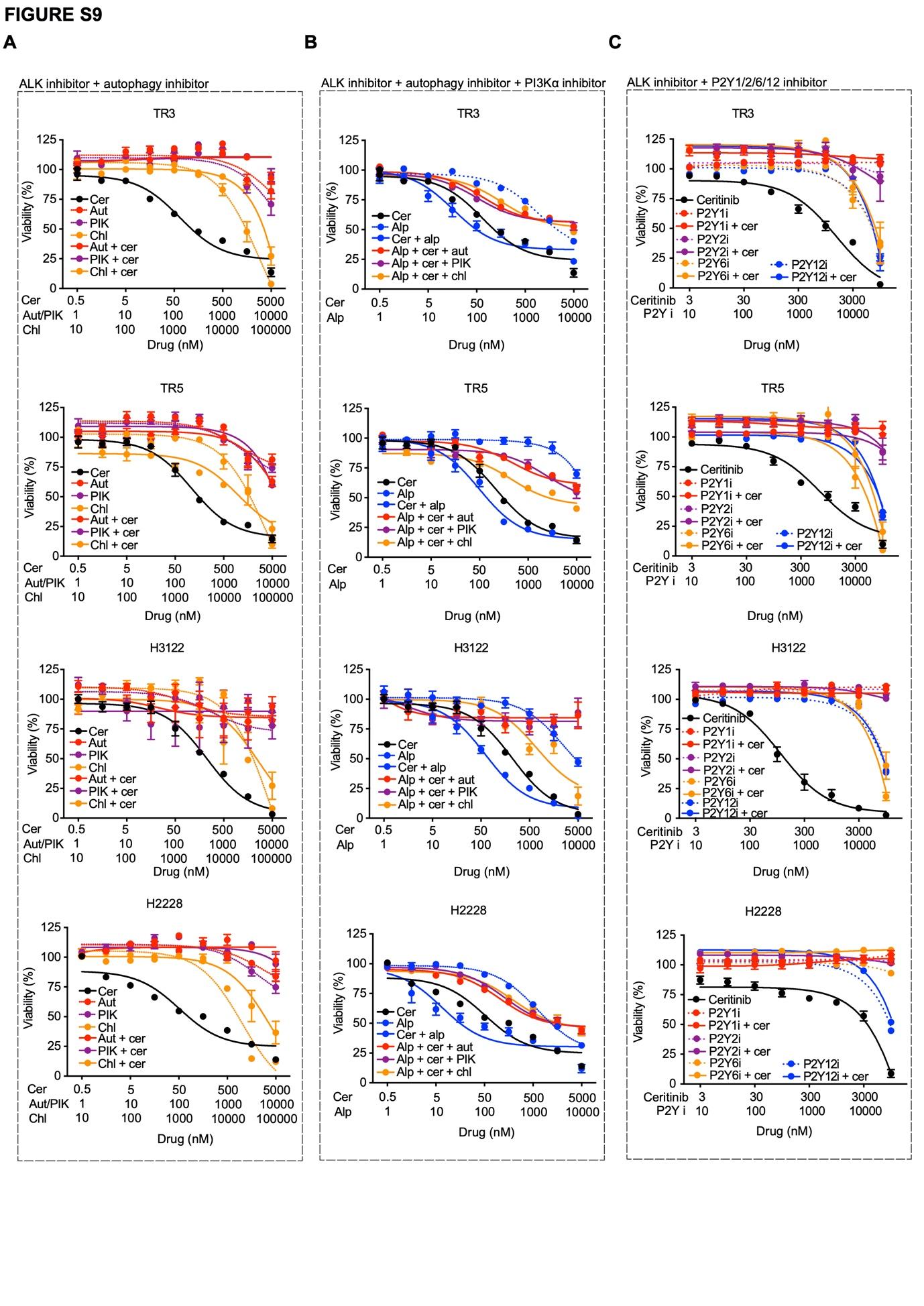
**

**Figure S9. Inhibition of autophagy or P2Y receptors does not improve the response of ceritinib.** (A) Dose-response curves of TR3, H2228 and H3122 cells treated with (A) ceritinib (cer), autophinib (aut; Vps34i), PIK-III (PIK; Vps34i), chloroquine (chl; autophagy inhibitor) or combinations of ceritinib plus 300 nM autophinib/PIK-III/chloroquine, (B) ceritinib, alpelisib (alp) or alpelisib in combination with 300 nm ceritinib or alpelisib in combination with 300 nm ceritinib plus 300 nM autophinib or 300 nM PIK-III or 10 μM chloroquine and (C) ceritinib, MRS2179 (P2Y1i), AR-C118925XX (P2Y2i), MRS2578 (P2Y6i), ticagrelor (tica; P2Y12i), or combination of P2Y inhibitors plus 200 nM ceritinib. Error bars represent ± SEM.

**
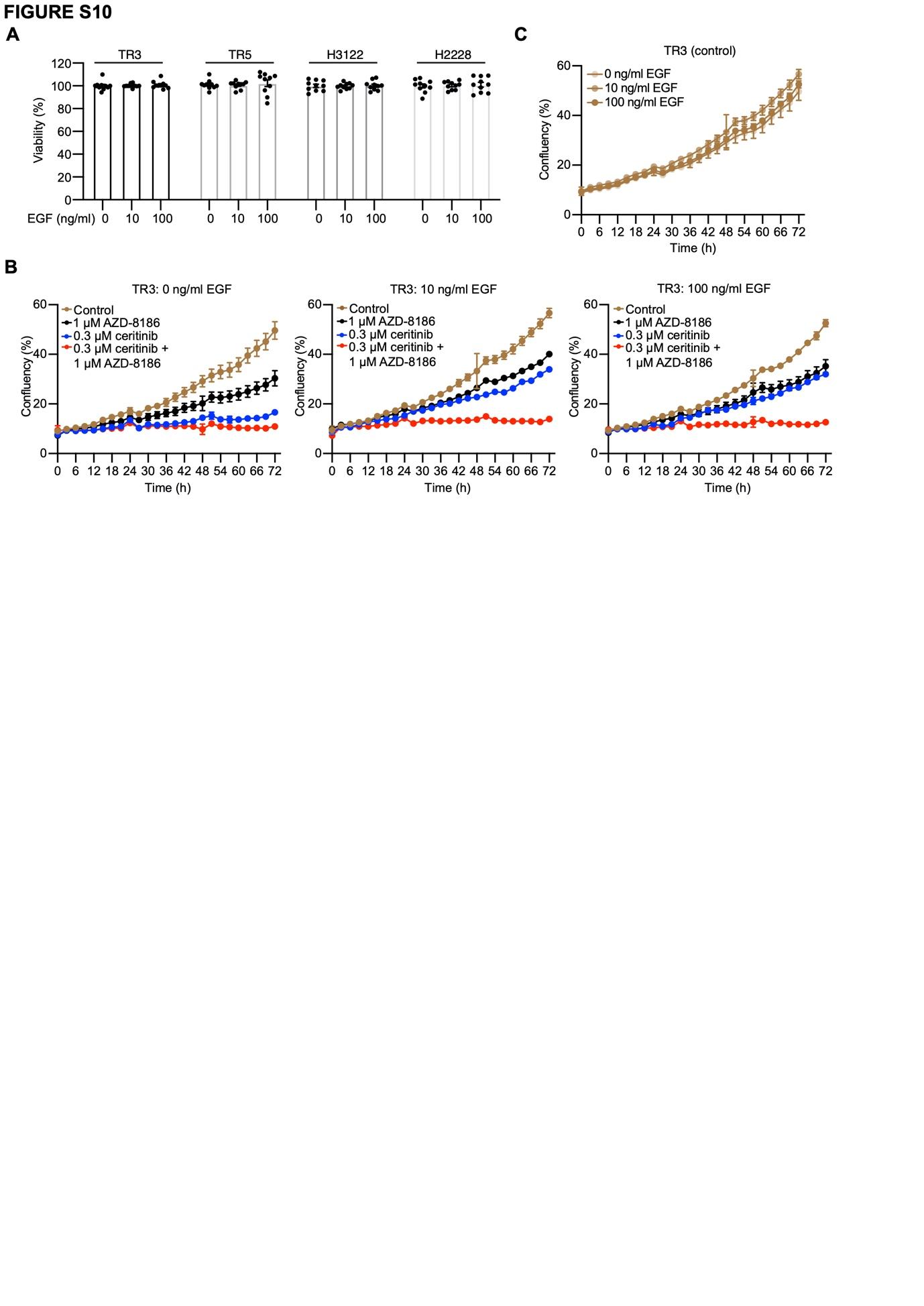
**

**Figure S10. Combined inhibition of ALK and PI3Kβ overcomes EGFR-mediated resistance in *ALK*-rearranged lung cancer cells.** (A) Cell viabilities of TR3, TR5, H3122 and H2228 DMSO control cells are treated with different doses of EGF (0, 10, and 100 ng/ml). (B) TR3 cells were co-treated with different doses of EGF (0, 10, and 100 ng/ml), vehicle (DMSO), ceritinib, AZD-8186, or the combination of ceritinib plus AZD-8186; cell confluency was measured every 3 h for 72 h using the Incucyte live cell imaging system. (C) TR3 control (DMSO) cells were treated with different doses of EGF (0, 10, and 100 ng/ml); cell confluency was measured every 3 h for 72 h using the Incucyte live cell imaging system.

**Table S14. Details of primary antibodies used in immunohistochemistry and western blotting analyses**

| **Immunohistochemistry** | | | |
| --- | --- | --- | --- |
| **Antibody** | **Company** | **Catalog no. (clone)** | **Antigen retrieval** |
| NKX2-1 | Abcam | ab133638 (EPR8190-6) | 10 mM sodium citrate (pH 6.0) |
| LKB1 | Cell Signaling Technology | 13031 (D60C5F10) | 10 mM sodium citrate (pH 6.0) |
| Ki-67 | Thermo Fisher  Scientific | RM-9106-S0 (SP6) | 10 mM sodium citrate (pH 6.0) |
| E-cadherin | Cell Signaling Technology | 3195  (24E10) | 10 mM sodium citrate (pH 6.0) |
| Pan-Cytokeratin (pan-CK) | Ventana | 760-2595 (AE1/AE3/PCK26) | ULTRA Cell Conditioning Solution (CC1) from Ventana (pH 8.6) |
| Cytokeratin 18 (CK 18) | Dako | M7010 (DC-10) | ULTRA Cell Conditioning Solution (CC1) from Ventana (pH 8.6) |
| Vimentin | Abcam | ab92547 (EPR3776) | 10 mM sodium citrate (pH 6.0) |
| CD31 | Abcam | ab28364 (Rabbit polyclonal) | Tris-EDTA (pH 9.0) |
| Collagen IV | Roche | 760-2632 (CIV22) | Cell Conditioning Solution (CC2) from Ventana (pH 8.6) |
| **Immunoblotting** | | | |
| **Antibody** | **Company** | **Catalog no. (clone)** | **Dilution/concentration** |
| α-Tubulin | Cell Signaling Technology | 2125 | 1:1000 |
| pAKT (Ser473) | Cell Signaling Technology | 4058 | 1:1000 |
| AKT | Cell Signaling Technology | 2920 | 1:1000 |
| pERK (Thr202/Tyr204) | Cell Signaling Technology | 4370 | 1:1000 |
| ERK | Cell Signaling Technology | 9107 | 1:1000 |
| pALK (Tyr1604) | Cell Signaling Technology | 3341 | 1:1000 |
| ALK | Cell Signaling Technology | 3633 | 1:1000 |
| pEGFR (Tyr1068) | Cell Signaling Technology | 3777 | 1:1000 |
| EGFR | Cell Signaling Technology | 4267 | 1:1000 |
| PI3Kβ | Cell Signaling Technology | 3011 | 1:1000 |
| Cleaved PARP (Asp214) | Cell Signaling Technology | 9541 | 1:1000 |
| **EGFR co-immunoprecipitation** | | | |
| **Antibody** | **Company** | **Catalog no. (clone)** | **Amount** |
| EGFR | Santa Cruz Biotechnology | sc-120 | 1 μg |

**Table S2**

**Table S3** *(Provided separately as an excel file)*

**Table S4**
